# PKMζ-PKCι/λ double-knockout demonstrates atypical PKC is crucial for the persistence of hippocampal LTP and spatial memory

**DOI:** 10.7554/eLife.110499

**Published:** 2026-07-22

**Authors:** Panayiotis Tsokas, Changchi Hsieh, Alejandro Grau-Perales, Andrew Tcherepanov, Leo Kwok, Laura Rodriguez-Valencia, David A Cano, Kim Allen, Hannah J Smith, Sabina Kubayeva, Benson J Wei, Samuel Sabzanov, Rafael Flores-Obando, Sourav Ghosh, Peter John Bergold, Jerry Rudy, James Cottrell, André Fenton, Todd Charlton Sacktor

**Affiliations:** 1 Department of Physiology and Pharmacology, The Robert F. Furchgott Center for Neural and Behavioral Science, State University of New York Downstate Health Sciences University Brooklyn United States; 2 Department of Anesthesiology, State University of New York Downstate Health Sciences University Brooklyn United States; 3 Department of Pathology, State University of New York Downstate Health Sciences University Brooklyn United States; 4 Center for Neural Science, New York University New York United States; 5 College of Medicine, State University of New York Downstate Health Sciences University Brooklyn United States; 6 Department of Biology, City University of New York-Medgar Evers College Brooklyn United States; 7 Departments of Neurology and Pharmacology, Yale University New Haven United States; 8 Department of Neurology, State University of New York Downstate Health Sciences University Brooklyn United States; 9 Department of Psychology and Neuroscience, University of Colorado at Boulder Boulder United States; 10 Neuroscience Institute at NYU Langone Medical Center New York United States; University of Texas at Austin United States; University of Texas at Austin United States

**Keywords:** PKMζ, PKMzeta, PKM-zeta, PKCι/λ, PKCι, PKCiota, PKCλ, PKClambda, Mouse

## Abstract

PKMζ is a persistently active atypical PKC (aPKC) isoform thought to maintain late-phase long-term potentiation (late-LTP) and long-term memory. PKMζ-knockout mice, however, still exhibit hippocampal LTP and spatial memory while lacking neocortical LTP, questioning whether this kinase is fundamental to enduring synaptic potentiation and memory. Tsokas et al. (2016) suggested that the other aPKC, PKCι/λ, may compensate for PKMζ during maintenance in the hippocampus of PKMζ-null mice. In wild-type mice, PKCι/λ drives early-LTP and short-term memory, whereas in PKCι/λ-knockout mice, PKMζ compensates by supporting both early- and late-phase processes. Here, we show that PKCι/λ is persistently upregulated during maintenance in two mouse models: PKMζ-conditional knockout mice, and double-knockout mice carrying both conditional deletion of PKCι/λ and constitutive loss of PKMζ. Because PKCι/λ-gene excision is inducible in the double-knockout line, we could characterize the persistent increase of PKCι/λ in late-LTP prior to its deletion. To examine PKCι/λ function, we induced its deletion in the hippocampus. Whereas mutual compensation preserves LTP when either PKCι/λ or PKMζ alone is knocked out, double-knockout of both PKCι/λ and PKMζ eliminates late-LTP. Double-knockout also abolishes spatial long-term memory without affecting short-term memory. Thus, when PKMζ is absent, PKCι/λ persists to maintain hippocampal late-LTP and long-term memory.

## Introduction

In PKMζ-knockout mice (PKMζ-KO), the maintenance of hippocampal LTP and long-term memory appears normal, and LTP is still disrupted by the atypical PKC (aPKC) inhibitor ZIP (Lee et al., 2013; Volk et al., 2013; Tsokas et al., 2016). In the same knockout mice, however, LTP is eliminated in medial prefrontal cortex (mPFC) (Kniffin et al., 2026; Sacktor, 2026). These results seem inconsistent, suggesting that the persistently active PKMζ is necessary only for maintaining LTP in mPFC, and not in hippocampus. But an alternative hypothesis is that compensation by another ZIP-sensitive maintenance molecule is induced in hippocampus by the absence of the gene for PKMζ (*Prkcz*). In Tsokas et al., 2016, multiple members of the PKC gene family were found to increase expression in compensation for the loss of PKMζ. The most promising candidate was the other aPKC, the ZIP-sensitive PKCι/λ (referred to as PKCι), since closely related genes can compensate for one another (Gu et al., 2003; Conant and Wagner, 2004; White et al., 2013; El-Brolosy and Stainier, 2017). Notably, in wild-type (WT) mice PKCι is critical to the initial generation of the early phase of LTP and short-term memory, and PKMζ compensates for the conditional knockout of the PKCι gene (*Prkci*) to support both short- and long-term processes (Ren et al., 2013; Tsokas et al., 2016; Wang et al., 2016; Sheng et al., 2017). Perhaps LTP and memory are maintained by a persistent kinase after all — but not always PKMζ.

The notion that the persistence of PKCι is the maintenance mechanism responsible for late-phase LTP and long-term memory in PKMζ-knockout mice raises two questions: (1) Does PKCι persist in LTP and long-term memory in the absence of PKMζ, and (2) Does eliminating both aPKCs abolish enduring LTP and long-term memory? We addressed these questions using conditional and double-knockout transgenic mouse strategies.

## Results

**Figure 1.**
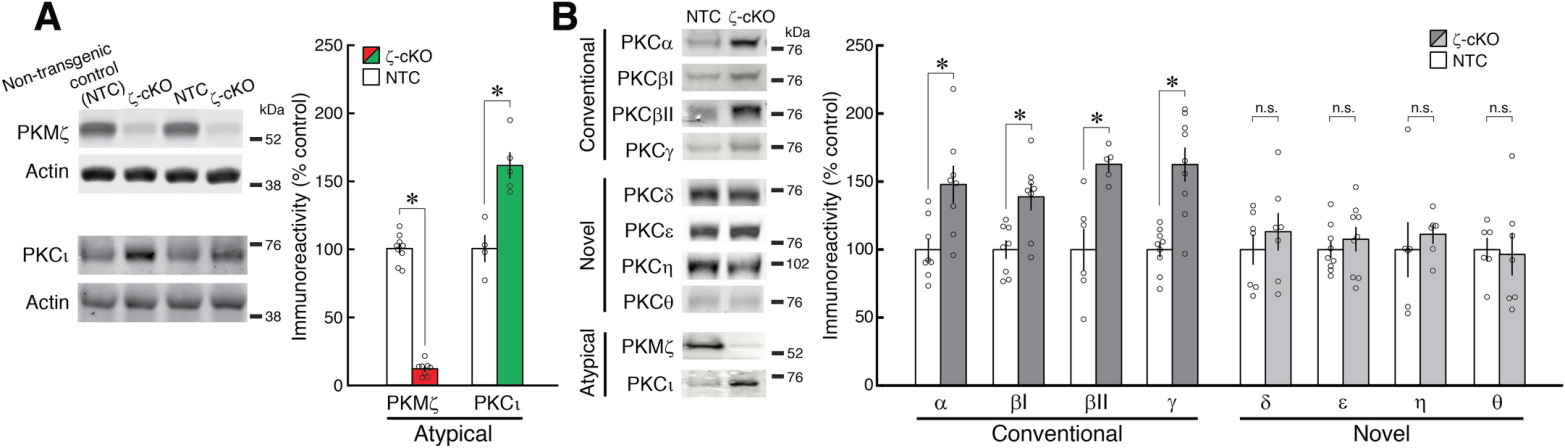
Compensatory increases of atypical PKCι and conventional, but not novel PKCs, in conditional PKMζ-knockout (ζ-cKO) mouse hippocampus. (**A, B**) Immunoblots of hippocampal extracts from *Camk2a-CreER^T2^; Prkcz^fl/fl^* mice that received tamoxifen (2 mg/200 µl i.p., five daily doses) to activate Cre recombinase selectively in excitatory neurons. Mice were sacrificed 7 days after the last dose. Left, representative immunoblots with M_r_ markers shown in kDa. Right, mean± SEM. Significance by two sample Student *t*-tests with Bonferroni correction denoted by *; not significant, n.s. Tamoxifen is a partial PKC antagonist and may still be present after a week (O’Brian et al., 1985); therefore, wild-type (WT) mice that also received tamoxifen are non-transgenic controls (NTC). (**A**) PKMζ decreases and PKCι increases in PKMζ-cKO mice. (**B**) Conventional PKCs increase and novel PKCs do not change. Actin loading controls shown in Figure 1—figure supplement 2. Statistics for (**A**) and (**B**) in Figure 1—source data 1. Figure 1—source data 1.Statistics for data presented in Figure 1. Figure 1—source data 2.Labeled immunoblots of Figure 1. Figure 1—source data 3.Unlabeled immunoblots of Figure 1.

As both inducible and constitutive PKMζ-KO mice still show hippocampal LTP (Volk et al., 2013), it is important to know if compensatory increases in other PKCs are present in the hippocampus of conditional PKMζ-KO mice (ζ-cKO), as was observed in PKMζ-null mice (Tsokas et al., 2016). In PKMζ-null mouse hippocampus, basal levels of PKCι and the conventional PKCβI increased (Tsokas et al., 2016). To examine the hippocampus of ζ-cKO mice, adult *Prkcz*-floxed mice expressing tamoxifen-inducible Cre recombinase under the Ca^2+^/calmodulin-dependent protein kinase IIα (CaMKIIα)-promoter (*Camk2a-CreER^T2^; Prkcz^fl/fl^* mice) were injected with tamoxifen. One week later, the expression of PKMζ in hippocampus decreased, and there was a compensatory increase in PKCι, as well as PKCι phosphorylated on its activation-loop (Figure 1A, Figure 1—source data 1, Figure 1—figure supplement 1A, Figure 1—figure supplement 1—source data 1). In addition, the expression of all four conventional PKCs (α, βI, βII, γ) increased, as did phosphorylation of the conventional PKC activation-loop (Figure 1B, Figure 1—source data 1, Figure 1—figure supplement 1A, Figure 1—figure supplement 1—source data 1, Figure 1—figure supplement 2). In contrast, there was no change in the expression of the novel PKC isoforms (δ, ε, η, θ), or phosphorylation of the activation-loop of PKCε (Figure 1B, Figure 1—source data 1, Figure 1—figure supplement 1A, Figure 1—figure supplement 1—source data 1, Figure 1—figure supplement 2). There was also no change in either the level of CaMKIIα or CaMKIIα T286-autophosphorylation, which initiates Ca^2+^-independent autonomous kinase activity (Miller and Kennedy, 1986) and LTP induction (Bayer and Giese, 2025; Tullis et al., 2023; Figure 1—figure supplement 1B, Figure 1—figure supplement 1—source data 1). Thus, both conditional and constitutive PKMζ-KO mice express compensatory increases in PKCι, as well as other PKCs, in hippocampus.

**Figure 1—figure supplement 1.**
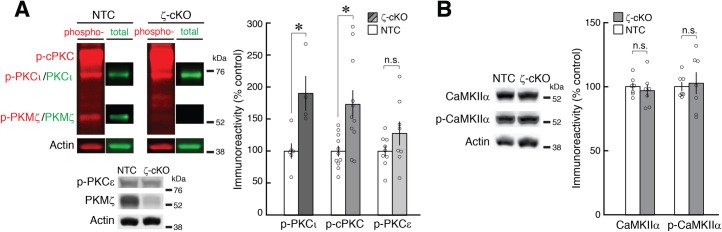
ζ-cKO increases the amounts of activation-loop-phosphorylated forms of atypical PKCι and conventional PKC, but not phosphorylated novel PKCε or T286-autophosphorylated CaMKIIα. (**A**) Left, above, representative immunoblots show increased amounts of activation-loop-phosphorylated PKCι (p-PKCι) and activation-loop-phosphorylated conventional PKC (p-cPKC) in PKMζ-cKO mice, compared to non-transgenic controls (NTC). Red, phospho-PKCs; green, total PKCs from the same samples. M_r_ markers shown at right. Below, activation-loop-phosphorylated p-PKCε (p-PKCε) recognized by its higher M_r_, does not change. Right, mean± SEM (**B**) Levels of total CaMKIIα and T286-autophosphorylated CaMKIIα do not change in ζ-cKO hippocampus. Statistics in Figure 1—figure supplement 1—source data 1. Figure 1—figure supplement 1—source data 1.Statistics for data presented in Figure 1—figure supplement 1. Figure 1—figure supplement 1—source data 2.Labeled immunoblots of Figure 1—figure supplement 1. Figure 1—figure supplement 1—source data 3.Unlabeled immunoblots of Figure 1—figure supplement 1.

**Figure 1—figure supplement 2.**
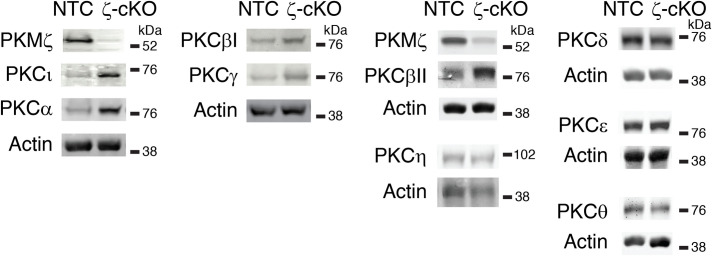
Actin loading controls for immunoblots shown in Figure 1B. The two left columns are from a pair of samples (non-transgenic control, NTC and ζ-cKO) processed on two mini-blots run in parallel. The third column is from a different pair of samples processed on two mini-blots run in parallel. The fourth column is from a third pair of samples processed on three mini-blots run in parallel.

**Figure 2.**
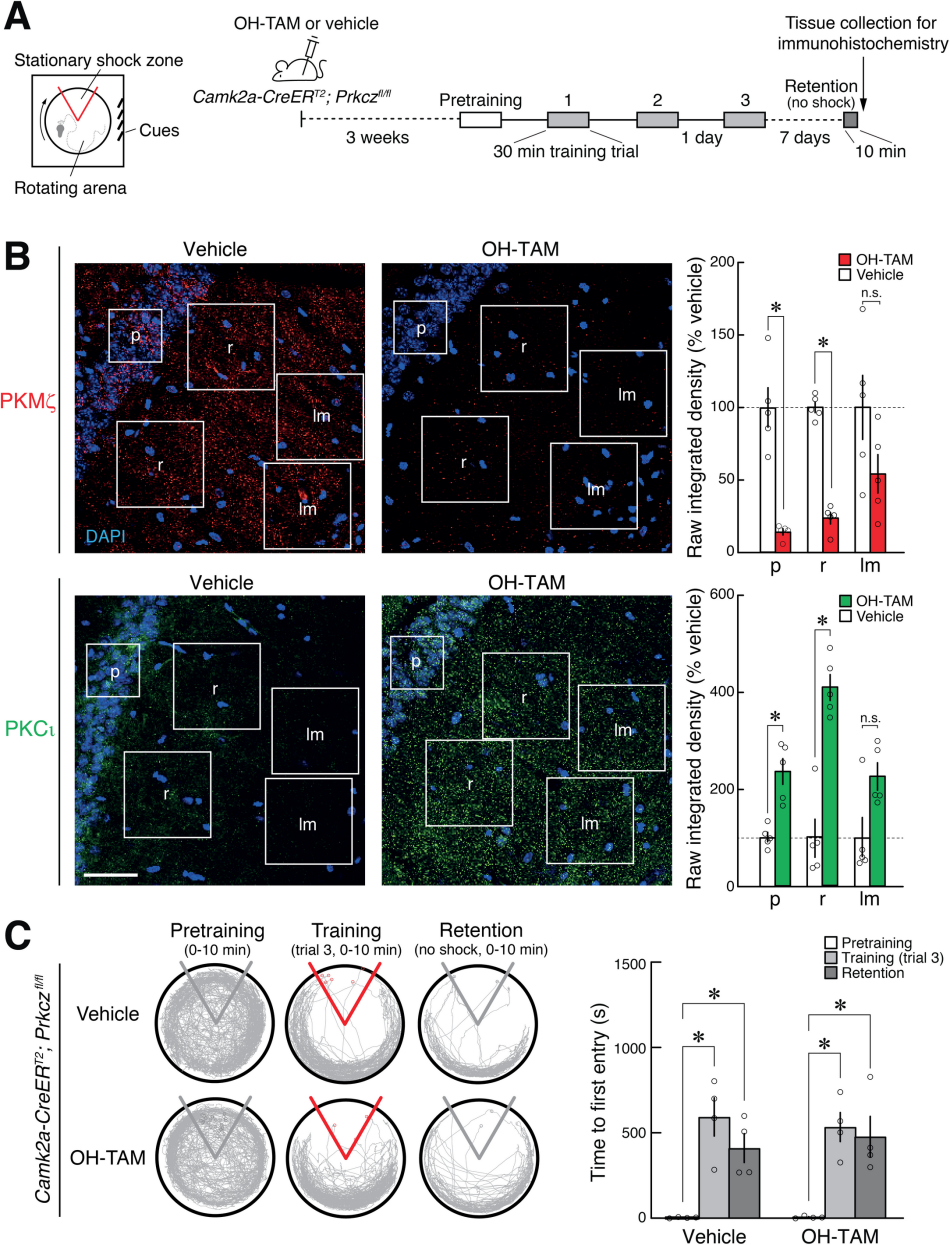
Compensatory increases of PKCι during spatial memory in conditional PKMζ-knockout (ζ-cKO) mouse hippocampus. (**A**) Left, schematic of active place avoidance training apparatus with a slowly rotating arena containing a nonrotating shock zone sector (shown in red). Visual cues located on the walls of the room are needed to avoid the shock zone. Right, experimental protocol. PKMζ is genetically ablated in *Camk2a-CreER^T2^; Prkcz^fl/fl^* mice. Cre is activated using 4-OH tamoxifen (OH-TAM, 2 mg/200 µl i.p., one injection every other day for three doses). Control mice receive vehicle injections. Active place avoidance training begins 3 weeks later, and 1 week after training memory retention is tested in the absence of shock followed immediately by sacrifice and immunohistochemistry. (**B**) Immunohistochemistry shows ζ-cKO reduces PKMζ and increases PKCι in CA1 *str. pyramidale* (**p**) and *radiatum* (**r**), but not *lacunosum-moleculare* (**lm**) 1 week after training. Left above, PKMζ expression decreases in cell bodies and dendritic compartments of the PKMζ-cKO. Left below, PKCι expression increases in cell bodies as well as in dendritic compartments where it is ordinarily expressed at relatively low levels. DAPI staining of nuclei shown in blue. Bar = 50 µm. Right, mean± SEM. Student *t*-tests with Bonferroni corrections compared differences in PKMζ and PKCι expression separately in the CA1 *strata* (Figure 2—source data 1). (**C**) Compensatory spatial memory in ζ-cKO. Left, representative paths during first 10 min of pretraining, training trial 3, and 1 day memory retention. Right, mean± SEM. Two-way ANOVA (treatment X training) revealed a significant effect of training (*F*_1.662, 9.972_ = 41.93, *p*<0.0001), but not an effect of treatment (*F*_1, 6_ < 0.001, *p*=1.0) or their interaction (*F*_2, 12_ = 0.48, *p*=0.6). Comparisons using Bonferroni-corrected tests revealed significant differences between pretraining and training (Trial 3) (*p*=0.002) and pretraining and retention (*p*=0.001), but no differences between training (Trial 3) and retention (*p*=0.1). Further comparisons of training in each treatment group separately revealed significant differences between pretraining and training (Trial 3) in both vehicle and 4-OH tamoxifen groups (*p*=0.02 and *p*=0.01, respectively) and between pretraining and retention in both vehicle and 4-OH tamoxifen groups (*p*=0.03 and *p*=0.05, respectively), confirming the treatment groups did not behave differently. Figure 2—source data 1.Statistics for data presented in Figure 2B.

Does the increase in PKCι persist in long-term memory, such that one aPKC substitutes for the other? Previous research has shown that PKMζ expression in CA1 *stratum (str.) radiatum* remains elevated for at least a month after training transgenic mice on a spatial memory task, as a component of a PKMζ-engram that traces the tri-synaptic circuit in the dendritic compartments of memory-tagged neurons (Hsieh et al., 2021; Han et al., 2026). We determined if PKMζ-cKO mice would also exhibit a compensatory increase of PKCι in *str. radiatum*. The PKMζ gene was ablated in adult *Camk2a-CreER^T2^; Prkcz^fl/fl^* mice, and 3 weeks later the mice were trained on the active place avoidance task to produce spatial memory (Figure 2A and C). One week after training, compared to vehicle-injected littermates, the PKMζ-cKO mice showed a decrease of PKMζ expression to ~25% and an increase of PKCι expression to ~400% (Figure 2B, Figure 2—source data 1). Thus, ζ-cKO mice after spatial training express compensatory, persistent increased expression of hippocampal PKCι.

**Figure 3.**
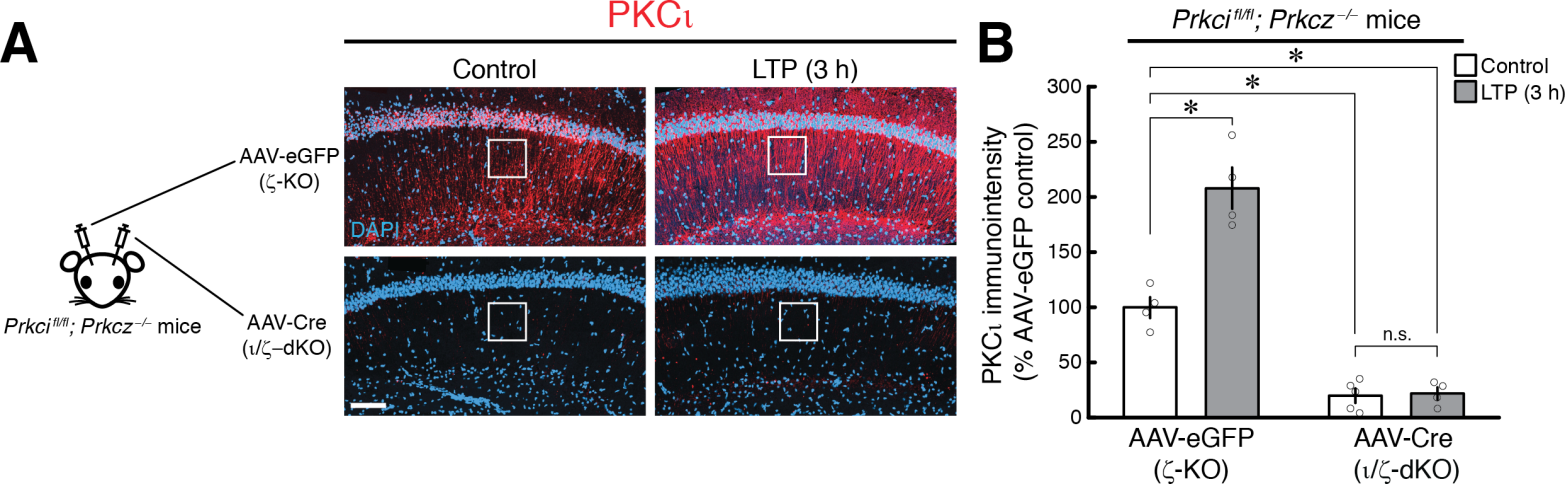
Compensatory increases of PKCι during hippocampal long-term potentiation (LTP) maintenance in *Prkci^fl/fl^; Prkcz^–/–^* mice. (**A**) Left, schematic shows adeno-associated virus (AAV) expressing Cre by cytomegalovirus (CMV) promoter injected into one hippocampus of a *Prkci^fl/fl^; Prkcz^–/–^* mouse, and control AAV expressing eGFP by CMV promoter injected into the contralateral hippocampus. Hippocampal slices are prepared 3 weeks later. Right, representative images of PKCι-immunohistochemistry. Top row, adjacent slices from AAV-eGFP-injected (ζ-KO) hippocampus show PKCι persistently increases 3 h post-tetanization. Bottom row, adjacent slices from the AAV-Cre-injected (ι/ζ-dKO) hippocampus show low levels of PKCι that do not change post-tetanization. White boxes show *str. radiatum* regions of interest. DAPI staining of nuclei shown in blue. Bar = 100 µm. (**B**) Mean± SEM. The two-way ANOVA reveals the main effects of treatment (AAV-Cre [ι/ζ-dKO] vs. AAV-eGFP [ζ-KO], *F*_1,13_ = 154.61, *p*<0.0001, *η*^2^_p_ = 0.92), and stimulation (high-frequency stimulation [HFS] vs. test stimulation, *F*_1,13_ = 26.61, *p*=0.0002, *η*^2^_p_ = 0.67), and an interaction of treatment X stimulation (*F*_1,13_ = 24.78, *p*=0.0003, *η*^2^_p_ = 0.66). *Post-hoc* analysis confirms that, compared to the ζ-KO control group, the intensity of PKCι immunoreactivity was significantly decreased in ι/ζ-dKO (*p*’s<0.0006 for both control and LTP), and increased in ζ-KO after HFS (*p*=0.0002). Intensity of residual PKCι immunoreactivity did not change in the ι/ζ-dKO between the control and HFS groups (*p*=0.9). ζ-KO, n’s=4; ι/ζ-dKO test, n=5; ι/ζ-dKO HFS, n=4.

Does the persistent increased expression of PKCι functionally compensate for the loss of PKMζ in LTP and memory in PKMζ-KO mice? As PKCι-null mice are embryonically lethal (Seidl et al., 2013), we determined the functional significance of the compensatory increase of PKCι for LTP by injecting an adeno-associated virus (AAV) expressing Cre recombinase in one hippocampus of PKCι-cKO/PKMζ-null (*Prkci^fl/fl^; Prkcz^–/–^*) mice to produce a double-knockout (dKO) (Sheng et al., 2017; Figure 3A). The contralateral hippocampus was injected with a control AAV expressing enhanced green fluorescent protein (eGFP), and 3 weeks later ex vivo slices were prepared. PKCι decreased in the ipsilateral hippocampus to ~20% compared to the contralateral hippocampus (Figure 3A and B).

**Figure 4.**
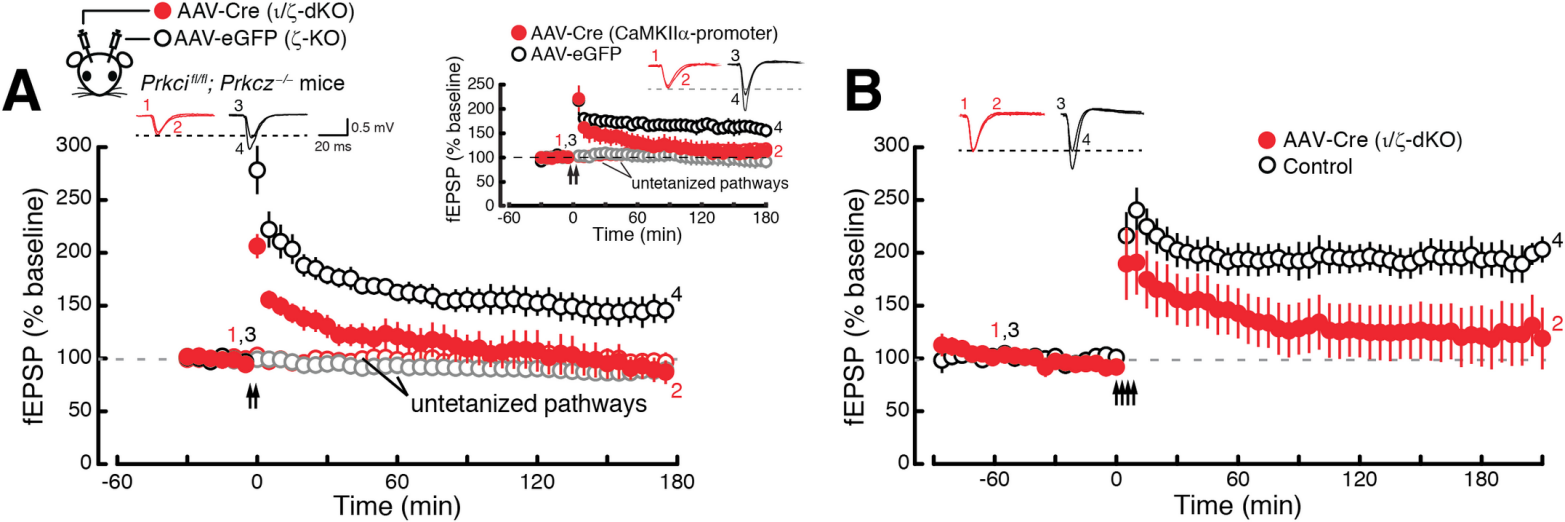
Impaired late-long-term potentiation (late-LTP) in ι/ζ-dKO hippocampus. (**A**) Late-LTP is absent in ι/ζ-dKO hippocampus. Above left inset, schematic of intracranial injections of adeno-associated virus (AAV)-Cre recombinase and AAV-eGFP into separate hippocampi of a *Prkci^fl/fl^; Prkcz^–/–^* mouse. Middle inset, color-coded representative field excitatory postsynaptic potentials (fEPSPs) correspond to numbered times in the time course below. Below, filled red circles, AAV expressing Cre by CMV promoter and high-frequency stimulation (HFS) with two tetanic trains; open red circles, test stimulation of a second synaptic pathway within the hippocampal slice. HFS tetani shown at arrows. Open black circles, AAV expressing eGFP by CMV promoter with HFS; open gray circles, with test stimulation. Three-way mixed-design ANOVA reveals main effects of treatment (hippocampal injections of AAV-Cre [ι/ζ-dKO] vs. AAV-eGFP [ζ-KO], *F*_1,20_ = 8.45, *p*=0.0009, *η*^2^_p_ = 0.30), and stimulation (HFS vs. test stimulation, *F*_1,20_ = 5.90, *p*=0.025, *η*^2^_p_ = 0.23), as well as a 3-way interaction among treatment X stimulation X time (5 min average of pre-HFS and 3 h post-HFS, *F*_1,20_ = 12.68, *p*=0.002, *η*^2^_p_ = 0.39). *Post-hoc* analysis confirms established LTP is not maintained in ι/ζ-dKO 3 h after HFS as compared to pre-HFS basal responses (*p*=0.7). *Post-hoc* analysis also confirms the control hippocampus maintains established LTP (*p*=0.0002). Test stimulation was unaffected by AAV-Cre or AAV-eGFP injections (*p*=0.9 and *p*=0.7, respectively). N’s=6. Right inset, ι/ζ-dKO by CaMKIIα promoter expression of Cre eliminates late-LTP. Three-way mixed-designed ANOVA reveals interaction between treatment (ζ-KO vs. ι/ζ-dKO) and stimulation (HFS vs. test stimulation, *F*_1,14_ = 6.62, *p*=0.02, *η*^2^_p_ = 0.32), and a 3-way interaction among treatment, stimulation, and time (5 min pre-HFS and 3 h post-HFS, *F*_1,14_ = 8.56, *p*=0.01, *η*^2^_p_ = 0.38). *Post-hoc* analysis confirms that compared to pre-HFS basal responses, LTP is not maintained in ι/ζ-dKO hippocampus 3 h post-HFS (*p*=0.8) and is maintained in the control hippocampus (*p*=0.003). Test stimulation was unaffected by AAV-Cre or AAV-eGFP injections (*p*=0.4 and *p*=0.9, respectively). ι/ζ-dKO HFS, n=5; ι/ζ-dKO test, n=4; ζ-KO HFS, n=5; ζ-KO test, n=4. (**B**) LTP does not persist in ι/ζ-dKO mice after stronger afferent stimulation with four tetanic trains. ANOVA with repeated measurements reveals main effects of time (5 min pre-HFS, 20 min post-HFS, and 3 h post-HFS, *F*_2,14_ = 20.51, *p*<0.0001, *η*^2^_p_ = 0.75). *Post-hoc* analysis confirms that early-LTP is established in both ι/ζ-dKO and control groups (5 min pre-HFS vs. 20 min post-HFS, *p*=0.005 and 0.002, respectively), and no difference between these two groups at 20 min post-HFS (*p*=0.6). However, LTP in ι/ζ-dKO did not persist 3 h (5 min pre-HFS vs. 3 h post-HFS, *p*=0.4), whereas LTP is intact in control (*p*=0.008). ι/ζ-dKO, n=5; control, n=4.

If PKCι is important for enduring LTP in PKMζ-KO mice, then this decrease should disrupt LTP persistence. High-frequency stimulation (HFS) of Schaffer collateral/commissural-CA1 synapses in the contralateral control slices, expressing PKCι but not PKMζ, induced persistent increases of the PKCι to ~200% and compensatory late-LTP, both lasting at least 3 h, the duration of the recordings (Figures 3 and 4A). In contrast, HFS of ipsilateral ι/ζ-dKO slices produced no persistent change in the residual PKCι and a transient LTP lasting only ~1–2 h (Figures 3 and 4A). Slices from the hippocampus of *Prkci^fl/fl^; Prkcz^–/–^* mice injected with AAV expressing Cre by the CaMKIIα-promoter to selectively ablate the PKCι gene in excitatory neurons resulted in similar transient LTP (Figure 4A, inset; Figure 4—figure supplement 1A). In addition, we showed that the virus injected into the hippocampus of *Prkci^fl/fl^; Prkcz^+/+^* mice, which express PKMζ, resulted in compensated early-LTP, as previously described (Sheng et al., 2017; Figure 4—figure supplement 1B). Thus, the individual knockout of each aPKC produces compensated LTP, whereas double-knockout of both aPKCs eliminates late-LTP.

**Figure 4—figure supplement 1.**
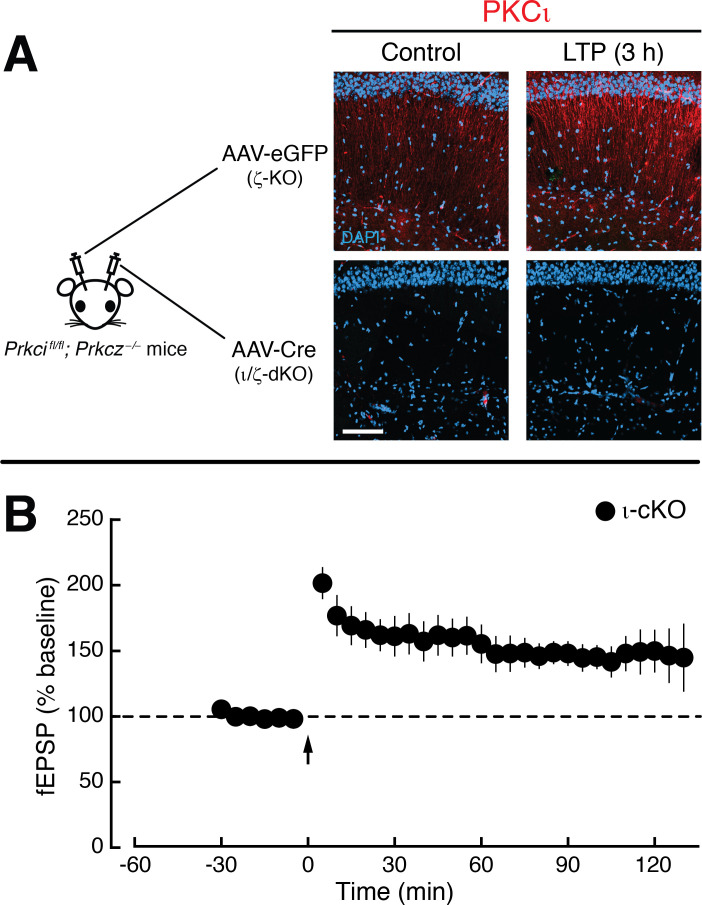
Effects of adeno-associated virus (AAV) expression of Cre recombinase by the CaMKIIα promoter in *Prkci^fl/fl^; Prkcz^–/–^* and *Prkci^fl/fl^; Prkcz^+/+^* mice. (**A**) Representative immunohistochemistry of AAV expressing Cre by CaMKIIα promoter in *Prkci^fl/fl^; Prkcz^–/–^* mice shows loss of PKCι in ι/ζ-dKO hippocampus and compensatory increase in PKCι during long-term potentiation (LTP) maintenance in control eGFP-injected hippocampus. Left, schematic of sites of injection; right, PKCι immunohistochemistry. DAPI staining of nuclei shown in blue. Bar = 100 µm. (**B**) ι-cKO (AAV with CaMKIIα promoter expressing Cre in hippocampus of *Prkci^fl/fl^; Prkcz^+/+^* mice) shows compensation of early-LTP, as previously described (Sheng et al., 2017). The repeated measurement ANOVA reveals the main effect of LTP (5 min average of pre-high-frequency stimulation (pre-HFS), 20 min post-HFS, and 2 h post-HFS, *F*_2,6_ = 14.03, *p*=0.005, *η*^2^_p_ = 0.82) in ι-cKO mice. *Post-hoc* tests confirm that LTP was established at 20 min post-tetanization (5 min pre-HFS vs. 20 min post-HFS, *p*=0.006) and maintained for 2 h (5 min pre-HFS vs. 120 min post-HFS, *p*=0.009); n=4.

We tested if late-LTP could be induced in ι/ζ-dKO hippocampus by increasing HFS from two trains, 20 s apart, which is optimized to produce an early onset of late-LTP (Tsokas et al., 2007), to four trains, spaced 5 min apart, which is optimized to produce maximal late-LTP (Scharf et al., 2002; Serrano et al., 2005; Figure 4B). The stronger stimulation induces LTP in ι/ζ-dKO slices that lasts only ~1–2 h. Three hours post-HFS, the field excitatory postsynaptic potentials (fEPSPs) were not significantly different from baseline fEPSPs before HFS.

**Figure 5.**
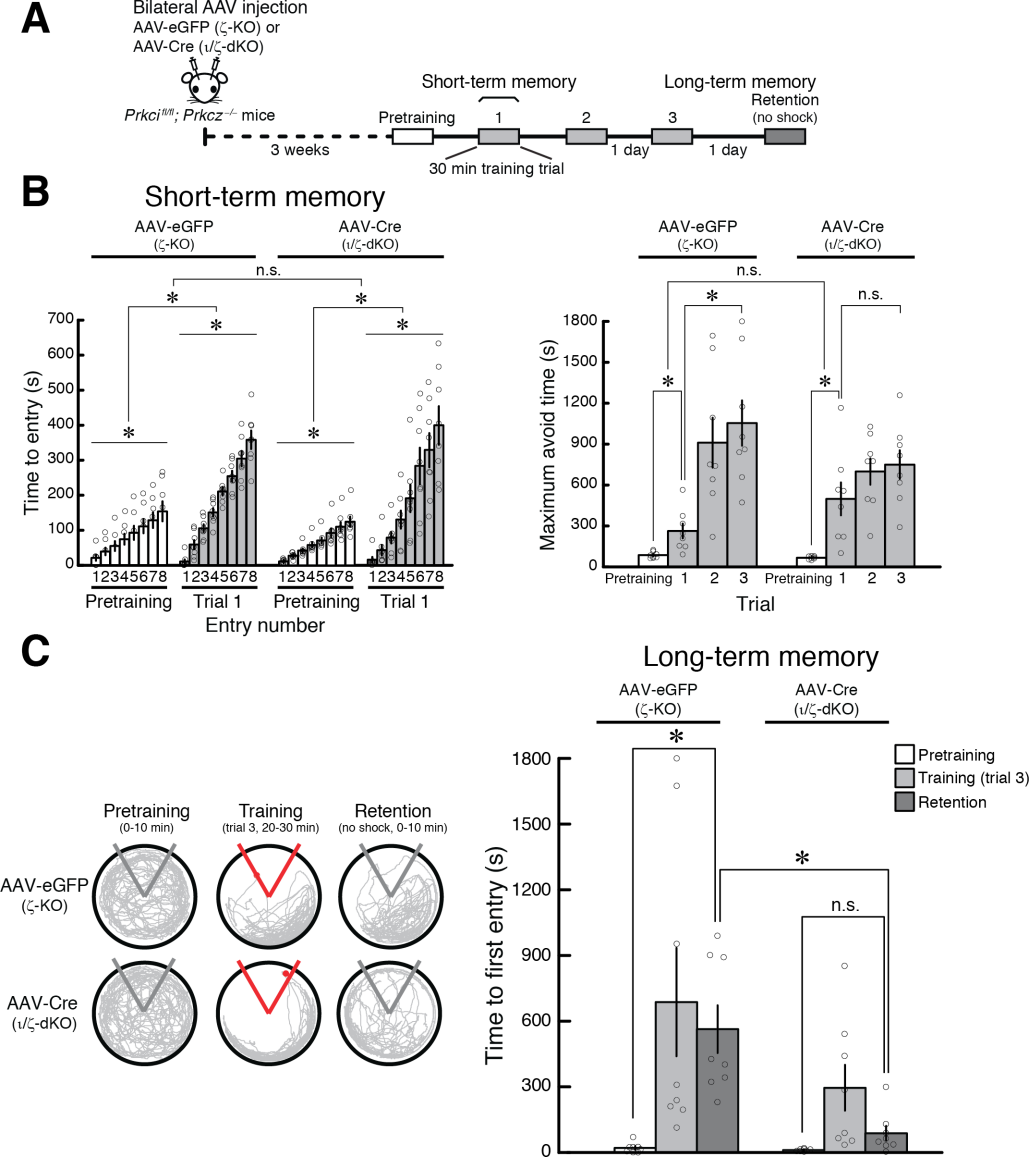
Impaired long-term memory and intact short-term memory for spatial information in mice with bilateral hippocampal ι/ζ-dKO. (**A**) Experimental protocol. *Prkci^fl/fl^; Prkcz^–/–^* mice are injected bilaterally in hippocampus with adeno-associated virus (AAV)-Cre (ι/ζ-dKO) or AAV-eGFP (ζ-KO, control), and 3 weeks later they received pretraining and, after 1 day, a single 30 min trial repeated daily for a total of three trials. Long-term retention is tested without shock 1 day after the last training trial. (**B**) ι/ζ-dKO does not affect short-term memory in the first training trial. Left, ι/ζ-dKO does not affect short-term memory as assessed by the time to enter the shock zone for the first eight entries (all animals had up to at least eight entries in trial 1). ANOVA with repeated measurements finds the main effects of training (pretraining and trial 1, *F*_1,28_ = 35.19, *p*<0.00001, *η*^2^_p_ = 0.56) indicating trial 1 learning, time to entry (the first to eighth entry within a trial, *F*_7,196_ = 145.80, *p*<0.00001, *η*^2^_p_ = 0.84), and their interaction (*F*_7,196_ = 37.68, *p*<0.00001, *η*^2^_p_ = 0.57). However, there is no group effect (AAV-eGFP- and AAV-Cre-injected, *F*_1,28_ = 0.19, *p*=0.7, *η*^2^_p_ = 0.007) nor interaction with either training or time to each entry (*F*’s<0.66, *p*’s>0.60, *η*^2^_p_’s<0.02). Right, ι/ζ-dKO does not affect short-term memory as assessed by maximum avoidance time during the first training trial. The contrast analysis reveals that the increases of maximum avoidance time from pretraining to trial 1 are not different between AAV-eGFP-injected and AAV-Cre-injected groups (*t*_14_=1.91, *p*=0.08, *d*=1.91). Paired *t*-tests reveal trial 1 is greater than pretraining in each genotype (*t*’s>3.10, *p*’s<0.018, Cohen’s *d*’s>1.62), indicating both groups of mice successfully established short-term memory. In contrast, the improvement of maximum avoidance time from trial 1 to trial 3 are different between the groups (*t*_14_=2.93, *p*=0.01, *d*=2.88), suggesting the two groups performed differently between daily training sessions when between-day memory influences avoidance. In addition, the ANOVA with repeated measurement discovers no group effect (AAV-eGFP-injected vs. AAV-Cre-injected, *F*_1,14_ = 0.56, *p*=0.47, *η*^2^_p_ = 0.04), but significant effects of trial (*F*_3,42_ = 30.37, *p*<0.0001, *η*^2^_p_ = 0.68), and interaction (*F*_3,42_ = 2.93, *p*=0.04, *η*^2^_p_ = 0.17). *Post-hoc* tests confirm that the maximum avoidance time in trial 1 is not different between the two groups (*p*=0.14). The AAV-eGFP-injected group improved its performance between trial 1 and trial 3 (*p*=0.0002), whereas the AAV-Cre-injected group showed no improvement (*p*=0.2; n’s=8). These data indicate no differences in short-term memory between AAV-eGFP- and AAV-Cre-injected groups, but only the AAV-Cre-injected failed to improve between daily trials, suggesting inability to retain avoidance memory across days. (**C**) PKCι gene ablation impairs long-term memory in *Prkci^fl/fl^; Prkcz^–/–^* mice. Left, representative paths during 10 min of pretraining, at end of training trial 3, and 1 day memory retention. Right, mean± SEM. The ANOVA with repeated measurement finds main effects of group (AAV-eGFP vs. AAV-Cre, *F*_1,14_ = 10.53, *p*=0.006, *η*^2^_p_ = 0.43) and training phase (pretraining, trial 3 of training, retention, *F*_2,28_ = 7.65, *p*=0.002, *η*^2^_p_ = 0.35). *Post-hoc* analysis reveals that the mice with AAV-Cre-injected ι/ζ-dKO hippocampus perform poorer during the memory retention test, compared to AAV-eGFP-injected littermates (*p*=0.02). The mice with ι/ζ-dKO hippocampus show no difference between the memory retention test and pretraining trial (*p*=0.9), whereas the AAV-eGFP-injected mice show long-term memory is maintained (*p*=0.02; n’s=8). In addition, pretraining vs. training trial 3 was significantly different in ζ-KO (*p*=0.006), but not in ι/ζ-dKO (*p*=0.4).

To determine if PKCι supports long-term memory in the absence of PKMζ, we injected *Prkci^fl/fl^; Prkcz^–/–^* littermates bilaterally in hippocampus with either AAV-Cre or AAV-eGFP, and then 3 weeks later compared spatial memory between the resulting ι/ζ-dKO and ζ-KO mice (Figure 5A). After a pretraining session without shock, the mice received three 30 min training trials separated by 24 h, and a final retention test without shock the next day. We assessed two measures of short-term memory (Figure 5B). First, we examined the time to each entry into the shock zone in the first training trial, as compared to the entries into the shock zone with the shock off during the pretraining session. This avoidance behavior increased within the first 30 min training session in both ι/ζ-dKO and ζ-KO mice, and the increases in the two genotypes were indistinguishable. Second, we measured the maximum avoidance time within each session. Maximum avoidance time reflects the time between shocks, which is controlled by both the animal’s memory within a trial and between trials, as well as its behavior. Compared to pretraining entries into the shock zone with the shock off, the maximum avoidance times for ζ-KO and ι/ζ-dKO mice increased in the first training trial. The increases in the first training trial were indistinguishable, indicating both genotypes acquired equivalent short-term memory for the shock zone. The maximum avoidance time for the ζ-KO, however, increased further over the 3 daily training sessions, whereas that of the ι/ζ-dKO did not, suggesting impaired long-term memory in the ι/ζ-dKO compared to the ζ-KO. Our main measure of long-term memory was time to first entry into the shock zone at the beginning of each session, which increases with memory maintained across days from previous trials (Figure 5C). The first entry times of the ζ-KO increased dramatically from pretraining to both session 3 and the retention test, indicating that mice with PKCι maintain long-term memory. In contrast, ι/ζ-dKO mice displayed a minimal increase at session 3 that was not significantly different from pretraining, and no difference between pretraining and the retention test, indicating loss of long-term memory.

## Discussion

Here, we found that in PKMζ-KO mice, the other aPKC, PKCι, becomes persistently active to maintain late-LTP and long-term memory. In WT mice, PKCι plays only a transient role in LTP and short-term memory (Ren et al., 2013; Tsokas et al., 2016; Wang et al., 2016; Sheng et al., 2017). In contrast, in both conditional and constitutive PKMζ-KO mice, the kinase is persistently upregulated during the maintenance of LTP and long-term memory (Figures 2 and 3). Because of their mutual compensation, hippocampal LTP is preserved when PKMζ or PKCι is knocked out individually. However, when both are knocked out, enduring LTP and long-term spatial memory are abolished (Figures 4 and 5, Figure 4—figure supplement 1B).

The ι/ζ-dKO exhibited an early transient synaptic potentiation that did not persist as late-phase LTP (Figure 4). Likewise, bilateral hippocampal ι/ζ-dKO did not prevent learning, short-term memory, or expression of place avoidance behavior, but eliminated spatial long-term memory (Figure 5). Because of PKCι’s contribution to early-LTP and short-term memory in WT mice (Ren et al., 2013; Wang et al., 2016), there must be additional molecules compensating for short-term processes when both aPKCs are genetically deleted. A candidate PKC isoform is the conventional PKCβI, which, like PKCι, is upregulated in both constitutive and conditional PKMζ-KO mice (Figure 1B; Tsokas et al., 2016).

Our finding that PKCι can substitute for PKMζ raises the question of how the compensation is induced. In knockout animals, Cre-mediated excision produces mRNA degradation fragments that can trigger compensatory gene expression (El-Brolosy and Stainier, 2017; El-Brolosy et al., 2019; Ma et al., 2019). This mechanism of compensation could explain how both constitutive and conditional PKMζ-KOs produce normal-appearing late-LTP/long-term memory, whereas PKMζ-shRNA and PKMζ-antisense oligodeoxynucleotides disrupt late-LTP/long-term memory when applied to WT mice, which retain full-length PKMζ mRNA transcription and lack compensation (Tsokas et al., 2016; Wang et al., 2016). In contrast to the hippocampus, both early- and late-LTP are eliminated in prefrontal cortex of PKMζ-KO mice (Kniffin et al., 2026). This finding suggests that the PKCι activation mediating early-LTP in the hippocampus of WT mice may not be present in prefrontal cortex to compensate for the loss of PKMζ (Sacktor, 2026).

Once increased, how does PKCι accomplish maintenance? The sustained action of PKMζ is driven by the isoform’s second messenger-independent, persistent enzymatic activity (Sacktor et al., 1993). PKMζ is autonomously active because the kinase is an independent catalytic domain that lacks the autoinhibitory PKCζ regulatory domain (Sacktor et al., 1993; Hernandez et al., 2003). PKCι, however, is a full-length PKC isoform with a regulatory domain that inhibits its catalytic domain. Therefore, for it to compensate for PKMζ, PKCι requires additional posttranslational mechanisms for persistent activation (Figures 2B and 3). PKCι can be activated by postsynaptic proteins, such as p62 that bind to its regulatory domain (Jiang et al., 2009; Ren et al., 2013). In contrast to the rapidly metabolized lipid second messengers that transiently stimulate PKCι and the conventional/novel PKCs, this protein-protein interaction may produce sustained PKCι kinase activity that can substitute for PKMζ.

Maintenance by PKMζ depends not only on its continuous activity but also on its continuous binding to the postsynaptic scaffolding protein KIBRA/WWC1 (kidney and brain protein/WW and C2 domain protein 1) (Tsokas et al., 2024; Shouval et al., 2025; Hsieh et al., 2026). This sustained interaction perpetually targets PKMζ to active synapses through persistent synaptic tagging, thus maintaining increases in the kinase despite protein turnover (Rudy, 2026). KIBRA also binds to PKCι, albeit more weakly than PKMζ (Tsokas et al., 2024). Therefore, in the hippocampus of WT mice, the strong binding of PKMζ to KIBRA could allow it to displace PKCι at active synapses in the transition from early- to late-LTP. In contrast, in the hippocampus of PKMζ-KO mice, PKCι would not be replaced by PKMζ. PKMζ and PKCι also compete for binding to PAR3 (partitioning defective protein 3), another postsynaptic protein that localizes aPKCs within neurons (Parker et al., 2013; Zhang and Wei, 2022). Thus, KIBRA and other synaptic tags that normally capture PKMζ in WT mice may, in the absence of PKMζ, persistently anchor PKCι to maintain hippocampus-dependent LTP and long-term memory.

Our findings reveal that the maintenance of synaptic potentiation and memory requires persistent aPKC activity, challenging the widely held view that synapses sustain memory through stable structural changes without specialized enzymes dedicated to information storage. The structural model, as introduced by Ramón y Cajal, hypothesized by Hebb, and established by Kandel (Ramón y Cajal, 1894; Hebb, 1949; Bailey and Kandel, 1993), has led to the identification of structural plasticity and non-enzymatic molecules that support LTP and memory, including cytoskeletal actin and the components of perineuronal nets (Matus, 2000; Tsien, 2013). But to address the fundamental problem of how memory might persist despite continuous protein turnover, Crick, Lisman, and Schwartz proposed that a self-perpetuating enzymatic mechanism stores information at synapses (Crick, 1984; Lisman, 1985; Schwartz, 1993). The search for a persistent biochemical process that maintains LTP for hours and long-term memory for days focused on two protein kinases, CaMKII and PKMζ (Lisman, 2017; Sacktor and Fenton, 2018). The kinase action of CaMKII, however, is crucial for initiating but not perpetuating LTP and memory (Tullis et al., 2023; Bayer and Giese, 2025). In contrast, PKMζ under physiological conditions in WT mice, and PKCι in the compensation of PKMζ-KO mice, play essential roles in the maintenance of late-LTP and long-term memory, a mechanism that is molecularly distinct from induction (Pastalkova et al., 2006; Shema et al., 2007; Shema et al., 2011; Wang et al., 2016; Tsokas et al., 2024). Future work will be required to determine if persistent changes in synaptic structure are sustained by the ongoing aPKC activity that maintains long-term memory (Chen et al., 2014).

## Materials and methods

**Key resources table:** 

Reagent type (species) or resource	Designation	Source or reference	Identifiers	Additional information
Strain, strain background (*Mus musculus*)	C57BL/6 J	The Jackson Laboratory	000664	
Strain, strain background (*Mus musculus,* C57BL/6)	*Prkcz^-/-^*	Messing Lab (Lee et al., 2013)		
Strain, strain background (*Mus musculus,* C57BL/6)	*Prkci^fl/fl^*	Ghosh Lab (El Ellam et al., 2024)		
Strain, strain background (*Mus musculus,* C57BL/6)	*Prkcz^fl/fl^*	Ghosh Lab (Mercau et al., 2024)		
Strain, strain background (*Mus musculus,* C57BL/6)	*Prkci^fl/fl^*; *Prkcz^-/-^*	Ghosh Lab (Scott et al., 2019)		
Strain, strain background (*Mus musculus*)	B6;129S6-Tg(Camk2a-cre/ERT2)1Aibs/J	The Jackson Laboratory	012362	
Genetic reagent (AAV2.9)	pENN.AAV.CMVs.PI.Cre.rBG	Addgene	105537	
Genetic reagent (AAV2.9)	pAAV.CMV.PI.EGFP.WPRE.bGH	Addgene	105530	
Genetic reagent (AAV2.9)	pENN.AAV.CamKII 0.4.Cre.SV40	Addgene	105558	
Antibody	PKMζ (C2) (Rabbit polyclonal)	Sacktor Lab (Hernandez et al., 2003)		WB (1:20,000) IHC (1:1000, Figure 2; 1:8000 to confirm ζ-null in other Figures)
Antibody	PKCι (E-7) (Mouse monoclonal)	Santa Cruz	sc-376344	WB (1:200) IHC (1:500)
Antibody	PKCι (C83H11) (Rabbit monoclonal)	Cell Signaling Technology	2998	WB (1:200) IHC (1:1000)
Antibody	PKCι (Mouse monoclonal)	BD Transduction Laboratories	610207	WB (1:200)
Antibody	Phospho-T410 PKCζ (H2) (Mouse monoclonal)	Santa Cruz	sc-271962	WB (1:200)
Antibody	Phospho-PKC (pan) (ζ Thr410) (190D10) (Rabbit monoclonal)	Cell Signaling Technology	2060	WB (1:100)
Antibody	Phospho-PKCζ (Thr410)/λ (Thr412) (Rabbit polyclonal)	Cell Signaling Technology	9378	WB (1:200)
Antibody	PKCα (H-7) (Mouse monoclonal)	Santa Cruz	sc-8393	WB (1:200)
Antibody	PKCα (Rabbit polyclonal)	Gibco	3191SA	WB (1:200)
Antibody	PKCβI (E-3) (Mouse monoclonal)	Santa Cruz	sc-8049	WB (1:500)
Antibody	PKCβII (F-7) (Mouse monoclonal)	Santa Cruz	sc-13149	WB (1:100)
Antibody	PKCβII (Rabbit polyclonal)	Sacktor Lab (Sacktor et al., 1993)		WB (1:100)
Antibody	PKCγ (C-4) (Mouse monoclonal)	Santa Cruz	sc-166385	WB (1:200)
Antibody	PKCγ (C-19) (Rabbit polyclonal)	Santa Cruz	sc-211	WB (1:500)
Antibody	PKCδ [EPR17075] (Rabbit monoclonal)	Abcam	ab182126	WB (1:100)
Antibody	PKCδ (G-9) (Mouse monoclonal)	Santa Cruz	sc-8402	WB (1:50)
Antibody	PKCε (E-5) (Mouse monoclonal)	Santa Cruz	sc-1681	WB (1:1000)
Antibody	PKCε (Rabbit polyclonal)	Messing Lab (Lee et al., 2013)		WB (1:1000)
Antibody	PKCη [EPR18513] (Rabbit monoclonal)	Abcam	ab179524	WB (1:200)
Antibody	PKCθ (E-7) (Mouse monoclonal)	Santa Cruz	sc-1680	WB (1:100)
Antibody	CaMKIIα (Mouse monoclonal)	ThermoFisher (Invitrogen)	137300	WB (1:100)
Antibody	p-CaMKII [T286] (Rabbit monoclonal)	Cell Signaling Technology	3361	WB (1:100)
Antibody	Actin (Mouse monoclonal)	Sigma	A4700	WB (1:5000)

### Reagents

Reagents were from MilliporeSigma unless otherwise stated. Antisera are listed in Key resources table.

### Animals

This study was performed in strict accordance with the recommendations in the Guide for the Care and Use of Laboratory Animals of the National Institutes of Health. All animals were handled according to approved Institutional Animal Care and Use Committee (IACUC) protocols (no. 11-10274, 15-10467) of the State University of New York (SUNY) Downstate Health Sciences University and protocol no. 15-1459 of New York University (NYU). The SUNY protocols were approved by the IACUC of SUNY Downstate Health Sciences University (animal welfare assurance number: D16-00167) and the NYU protocol was approved by the NYU Animal Welfare Committee (animal welfare assurance number: A3317-01). All surgery was performed under isoflurane anesthesia. All efforts were made to minimize animal suffering and to reduce the number of animals used. Animals were male mice on C57BL/6 background and at least 4-months-old for all experiments. The PKMζ-null mouse line was previously described (Lee et al., 2013) and provided by Robert O. Messing (Univ. Texas at Austin, TX, USA). Conditional PKMζ and PKCι mice were generated by Sourav Ghosh as previously described (El Ellam et al., 2024; Mercau et al., 2024; Scott et al., 2019). *Camk2a-CreER^T2^* mice were from Jackson Labs, and the vehicle for tamoxifen i.p. injections to induce gene deletion was sunflower seed oil.

### Hippocampal slice recording and stimulation

Acute mouse hippocampal slices (450 µm) were prepared as previously described (Tsokas et al., 2016; Tsokas et al., 2019). Hippocampi were dissected, bathed in ice-cold dissection buffer, and sliced with a McIlwain Tissue Chopper in a cold room at 4 °C. The dissection buffer contained (in mM): 125 NaCl, 2.5 KCl, 1.25 NaH_2_PO_4_, 26 NaHCO_3_, 11 glucose, 10 MgCl_2_, and 0.5 CaCl_2_, and was bubbled with 95% O_2_/5% CO_2_ to maintain pH at 7.4. After dissection, the slices were transferred to an Oslo-type interface recording chamber (31.5±1°C) (Tsokas et al., 2019). The recording superfusate consisted of (in mM): 118 NaCl, 3.5 KCl, 2.5 CaCl_2_, 1.3 MgSO_4_, 1.25 NaH_2_PO_4_, 24 NaHCO_3_, and 15 glucose, bubbled with 95% O_2_/5% CO_2_, with a flow rate of 0.5 ml/min.

Field EPSPs were recorded with a glass extracellular recording electrode (2–5 MΩ) placed in the CA1 *str. radiatum*, and concentric bipolar stimulating electrodes (CBBRE75 and 30200; FHC, Bowdoin, ME) were placed on either side within CA3 or CA1. Test stimulation rate was once every 30 s in each stimulating electrode, alternating every 15 s between electrodes. Based upon a pre-established exclusion criterion, a slice was not used if fEPSP spike threshold was <2 mV on initial input-output analysis. Pathway independence was confirmed by the absence of paired-pulse facilitation between the two pathways. A single stimulating electrode with a test stimulation rate of once every 30 s was used for immunohistochemistry experiments. HFS optimized to produce a relatively rapid onset of protein synthesis-dependent late-LTP consisted of two 100 Hz-1 s tetanic trains, at 25% of spike threshold, spaced 20 s apart (Tsokas et al., 2005). HFS optimized to produce maximal late-LTP consisted of four 100 Hz-1 s tetanic trains, at 25% of spike threshold, spaced 5 min apart (Scharf et al., 2002; Serrano et al., 2005). The maximum slope of the rise of the fEPSP was analyzed on a PC using the WinLTP data acquisition program (Anderson and Collingridge, 2007).

### Immunoblots and immunohistochemistry

Immunoblots of total hippocampus were performed as previously described (Tsokas et al., 2016), using antibodies in the Key resources table. To avoid mixing primary antisera from the same species, we adapted a method of cutting full-length nitrocellulose after transfer, prior to immunostaining (Sacktor et al., 1993). Using visible molecular weight markers, we cut the full-length nitrocellulose into horizontal sections that contained PKCs of different molecular weights. In the source data files, the actin loading control lanes of the full-length gel are shown under each horizontal section. PKCs with similar molecular weights were either stained with antisera from different species or examined on separate blots. Four damaged lanes whose densitometry could not be accurately measured were not included in the analysis and are marked in the source data with an ø. Immunoblot densitometry was performed with NIH ImageJ (version 1.53 a) using the Gel Analysis tool. Each band was measured in triplicate, and the average of these measurements was taken as representative of the density of the band. In a subset of immunoblots, we used the actin bands from Ponceau S staining as loading controls, as denoted in the source data files.

Immunohistochemistry for Figure 3 and Figure 4—figure supplement 1A was performed as previously described (Hsieh et al., 2021; Tsokas et al., 2024), using mouse anti-PKCι primary antibody (1:500, E-7, Santa Cruz SC-376344).

Methods for immunohistochemistry shown in Figure 2B were as follows. Free-floating sections were permeabilized with phosphate-buffered saline (PBS) containing 0.1% Tween-20 (PBS-T) for 1 h at room temperature and blocked with 10% normal goat serum in PBS-T (blocking buffer) for 2.5 h at room temperature. One batch of sections was incubated overnight at 4 °C with rabbit anti-PKMζ C-2 antisera primary antibody (1:1000) (Hernandez et al., 2003) and a second batch of sections with rabbit anti-PKCι (1:1000, Cell Signaling #2998) in blocking buffer. After washing three times for 10 min each in PBS-T, both batches of sections were incubated with the secondary antibody goat anti-rabbit-Alexa 647 (1:500 in blocking buffer; Jackson ImmunoResearch) for 2 h at room temperature. After washing three times for 10 min each in PBS-T and extensive washing with PBS, the sections were mounted with Vectashield with 4′,6-diamidino-2-phenylindole (DAPI, Vector Laboratories). Three sections from each mouse were examined using an upright Leica SP8 confocal microscope and analyzed using ImageJ (version 1.53 a). For each section, 8.5 µm-thick Z-stacks of the dorsal CA1 were created using the maximum intensity projection function in ImageJ. For each *str. pyramidale*, *radiatum*, and *lacunosum-moleculare*, two square regions of interest were centered in each stack. Measurements were made from each mouse in each region of interest. The raw integrated density (defined as the sum of the values for all pixels) of the Z-stack region of interest expressing the fluorescent label was measured for the volume of target pixels, and the average of each measurement was taken as representative for the region of each mouse.

### AAV injections

Mice were anesthetized in a closed chamber filled with the inhalation anesthetic isoflurane (RWD Life Science, R510-22-10) and then fixed in a stereotaxic apparatus (Stoelting Co.). Anesthesia was maintained with isoflurane inhalation (1–2.5% via trachea). The eyes of the mice were safeguarded using erythromycin ophthalmic ointment (0.5%). The skull was exposed and cleaned using 3% hydrogen peroxide. Small holes in the skull were then drilled with the following stereotaxic coordinates: left hippocampus (triple injection: AP: –1/ML: –0.7/DV: –1.65; AP: –1.8/ML: –1.5/DV: –2; AP: –2.7/ML: –2/DV: –2, below the skull surface) and right hippocampus (triple injection: AP: –1/ML:+0.7/DV: –1.65; AP: –1.8/ML:+1.5/DV: –2; AP: –2.7/ML:+2/DV: –2, below the skull surface). The virus was injected using a 34-gauge needle with a Hamilton syringe at 0.1 µl/min rate into target regions. At all injected points, the tip of the needle was positioned 0.05 mm below the target coordinate and returned to the target site after 2 min. After injection, the needle stayed in place for an additional 7 min and was slowly withdrawn. AAVs expressing Cre recombinase and eGFP were from Addgene. For physiology, 0.5 µl of virus pENN.AAV.CMVs.PI.Cre.rBG (AAV2.9) (1×10^13^ viral genomes [vg]/ml) was injected into one CA1, and 0.5 µl of virus pAAV.CMV.PI.EGFP.WPRE.bGH (AAV2.9) (1×10^13^ vg/ml) was injected in the contralateral side. For behavior, each virus was injected bilaterally in the hippocampus of individual animals. Virus pENN.AAV.CamKIIa 0.4 Cre SV40 (AAV2.9) (1×10^13^ vg/ml) was used to express Cre recombinase by the CaMKIIα promoter.

### Conditioning

Active place avoidance was conducted with a commercial computer-controlled system (Bio-Signal Group, Acton, MA). The mouse was placed on a 40 cm diameter circular arena rotating at 1 rpm. The specialized software, Tracker (Bio-Signal Group, Acton, MA), was used to detect the animal’s position 30 times per second by video tracking from an overhead camera. A clear wall made from polyethylene terephthalate, glycol-modified (PET-G) was placed on the arena to prevent the animal from jumping off the elevated arena surface. The arena was surrounded by opaque black curtains forming a 3 m X 4 m square, on which several distal visual landmarks were placed. A 5-pole shock grid was placed on the rotating arena, and the shock was scrambled across the five poles when the mouse entered the shock zone. All experiments used the ‘Room+Arena-’ task variant that challenges the mouse on the rotating arena to avoid a shock zone that was a stationary 60° sector (Pastalkova et al., 2006). Every 33 ms, the software determined the mouse’s position, whether it was in the shock zone, and whether to deliver shock. After the animal enters the shock zone for 500 ms, a constant current foot-shock (60 Hz, 500 ms) was delivered and repeated with an interval of 1500 ms until the mouse left the shock zone. The shock intensity was 0.2 or 0.3 mA, which was the minimum amplitude to elicit flinch or escape responses. The animal was forced to actively avoid the designated shock zone because the arena rotation periodically transported it into the shock area. A pretraining period on the apparatus without shock that was equivalent in time to a training session was provided.

The tracked animal positions with timestamps were analyzed offline (TrackAnalysis, Bio-Signal Group, Acton, MA) to extract several end-point measures. The time to first enter the shock zone estimates ability to avoid shock and was taken as an index of between-session long-term place avoidance memory. Short-term memory was assessed by two measures. First, the times to each entry into the shock zone in the first training trial were compared to the times for each entry into the shock zone with the shock off during the pretraining session. Time to enter the shock zone was examined for the first eight entries as all animals had up to at least eight entries in trial 1. Avoidance behavior is observed as an increase in the amplitude of the times for entering the shock zone. Second, the maximum time without receiving a shock was determined for each session. Short-term memory for avoidance behavior is measured as an increase in the maximum time between shocks in the first training trial, compared to the maximum time between entrances into the shock zone with the shock off during pretraining.

For Figure 5 the training schedule was as follows: 1 day after a 30 min pretraining session, the animals received three 30 min training trials, with an intertrial interval of 1 day. Long-term memory retention was tested the following day without shock. Pre-established exclusion criterion was if cannulae were found to be incorrectly targeted. No mice were excluded.

### Statistics

All experiments were performed with blind procedures except for LTP experiments that involved transfection with AAV-eGFP, as the eGFP could be detected visually in the hippocampal slice by the experimenter. Animals within a genotype were randomly assigned to the treatment groups. All data describe biological replicates. Sample sizes vary for the different experimental approaches (immunoblotting, immunohistochemistry, extracellular field potential physiology, and behavior). The hypothesis that PKMζ is compensated predicts all-or-none effects in the experiments, and this provided a basis for sample size estimates. Power analyses were performed using G*Power Version 3.1.9.7 with *α*=0.05 and *β*=0.8 and large effect sizes of 1.5–2.0. The effect size estimates were based on prior studies that demonstrated predominantly all-or-none effects of PKMζ inhibition on the immunoblot, immunohistochemical, physiological, and behavioral assays used here (Tsokas et al., 2016; Hsieh et al., 2021; Tsokas et al., 2024). For immunoblot densitometry and immunohistochemistry fluorescence values, Grubbs’ test was used to identify outliers for each experimental group; 5 out of 303 total values were thus excluded and denoted in the numerical data source file. Two-population Student *t* tests with Bonferroni corrections were performed to compare protein levels by immunoblot and immunohistochemistry in the PKMζ-cKO and control mice. For LTP experiments, the responses to test stimuli were averaged across 5 min for statistical comparisons. Repeated measures ANOVA was used to compare the change in the pre-tetanization and post-tetanization responses at the time points described. Multi-factor comparisons were performed using mixed-design ANOVA with repeated measures, as appropriate. The degrees of freedom for the critical *t* values of the *t* tests and the *F* values of the ANOVAs are reported as subscripts. *Post-hoc* multiple comparisons were performed by Newman-Keuls tests as appropriate. Statistical significance was accepted at *p*<0.05. Effect sizes for binary comparisons and one-way ANOVAs are reported as Cohen’s *d* and as *η*^2^_p_ for multi-factor ANOVA effects.

## Data Availability

Figures and tables contain all the data used to generate the figures and tables.

## Additional files

MDAR checklist

Source data 1. Numerical data for Figures 1—5, Figure 1—figure supplement 1, Figure 4—figure supplement 1B.
