# Supplementary material for: PKMζ-PKCι/λ double-knockout demonstrates atypical PKC is crucial for the persistence of hippocampal LTP and spatial memory
Source: eLife. 2026 Jul 22;15:RP110499. doi: 10.7554/eLife.110499 (PMC13391083; doi:10.7554/eLife.110499)
Supplement: Figure 1—source data 1. [file elife-110499-fig1-data1.docx]

|  | Non-transgenic control (NTC) | |  | ζ-cKO | |  |  |  |  |
| --- | --- | --- | --- | --- | --- | --- | --- | --- | --- |
| Isozyme | Mean ± SEM | n |  | Mean ± SEM | n | Degree of freedom | *t* | *P* | Cohen’s *d* |
| **(A**) |  |  |  |  |  |  |  |  |  |
| aPKC |  |  |  |  |  |  |  |  |  |
| **PKMζ** | **100 ± 3.5** | **9** |  | **11.8 ± 1.8** | **8** | **15** | **21.5** | **< 0.0001** | **10.4** |
| **PKCι** | **100 ± 9.5** | **4** |  | **160.8 ± 9.4** | **5** | **7** | **4.5** | **0.003** | **3.0** |
|  |  |  |  |  |  |  |  |  |  |
|  |  |  |  |  |  |  |  |  |  |
| **(B**) |  |  |  |  |  |  |  |  |  |
| cPKC |  |  |  |  |  |  |  |  |  |
| **α** | **100 ± 7.7** | **8** |  | **147.8 ± 13.2** | **8** | **14** | **3.12** | **0.008** | **1.6** |
| **βI** | **100 ± 6.5** | **8** |  | **138.7 ± 9.7** | **8** | **14** | **3.3** | **0.005** | **1.7** |
| **βII** | **100 ± 15.0** | **6** |  | **162.5 ± 5.5** | **5** | **9** | **3.6** | **0.006** | **2.2** |
| **γ** | **100 ± 5.3** | **9** |  | **162.4 ± 12.0** | **9** | **16** | **4.7** | **0.0002** | **2.2** |
|  |  |  |  |  |  |  |  |  |  |
| nPKC |  |  |  |  |  |  |  |  |  |
| δ | 100 ± 10.6 | 7 |  | 113.1 ± 13.3 | 7 | 12 | 0.8 | 0.5 | 0.4 |
| ε | 100 ± 5.7 | 9 |  | 107.7 ± 8.6 | 9 | 16 | 0.7 | 0.5 | 0.4 |
| η | 100 ±19.8 | 6 |  | 111.3 ± 6.7 | 6 | 10 | 0.5 | 0.6 | 0.3 |
| θ | 100 ± 8.4 | 6 |  | 96.6 ± 15.1 | 7 | 11 | 0.2 | 0.9 | 0.1 |

**Figure 1 — source data 1. Statistics for data presented in (A) Figure 1A and (B) Figure 1B.** Significant differences with Bonferroni correction are in bold.
