# Supplementary figures and images for: PKMζ-PKCι/λ double-knockout demonstrates atypical PKC is crucial for the persistence of hippocampal LTP and spatial memory
Source: eLife. 2026 Jul 22;15:RP110499. doi: 10.7554/eLife.110499 (PMC13391083; doi:10.7554/eLife.110499)

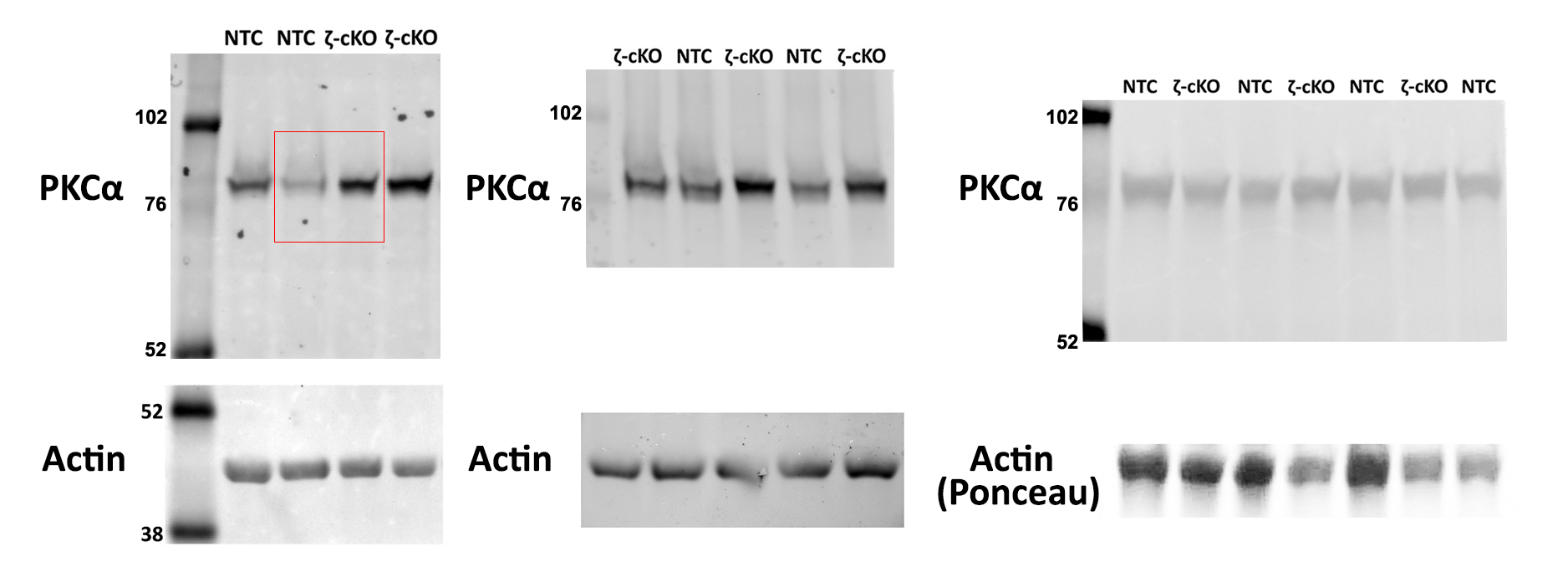

Supplement: Figure 1—source data 2. [file elife-110499-fig1-data2.zip › Figure 1B-Labeled/alpha/Composite PKCalpha Actin-Labeled.tif]

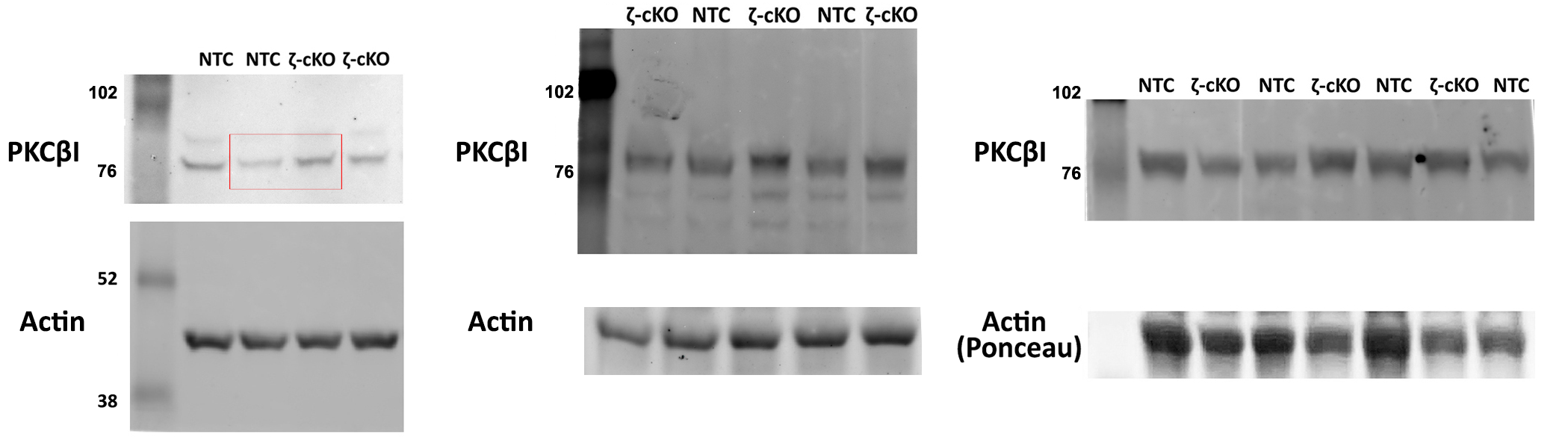

Supplement: Figure 1—source data 2. [file elife-110499-fig1-data2.zip › Figure 1B-Labeled/beta I/Composite PKCbetaI Actin-Labeled.tif]

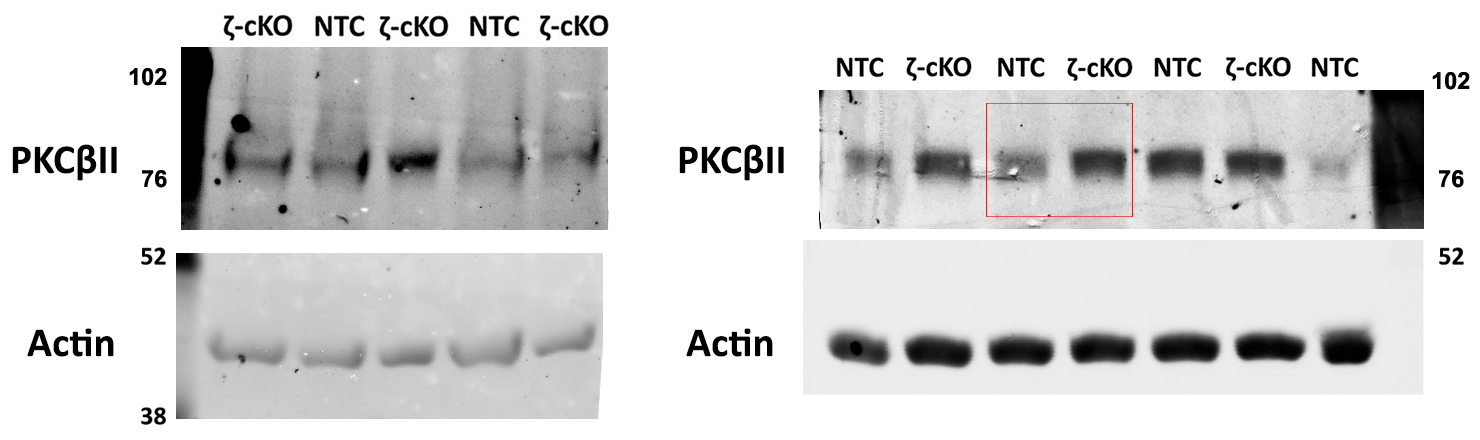

Supplement: Figure 1—source data 2. [file elife-110499-fig1-data2.zip › Figure 1B-Labeled/beta II/Composite PKCbetaII Actin-Labeled.tif]

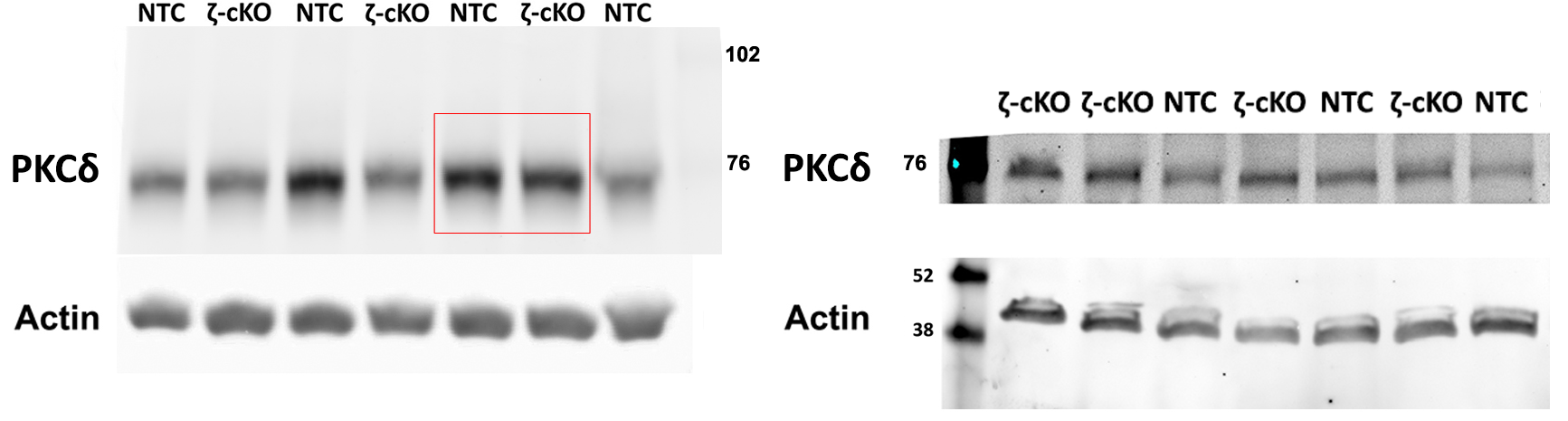

Supplement: Figure 1—source data 2. [file elife-110499-fig1-data2.zip › Figure 1B-Labeled/delta/Composite PKCdelta Actin-Labeled.tif]

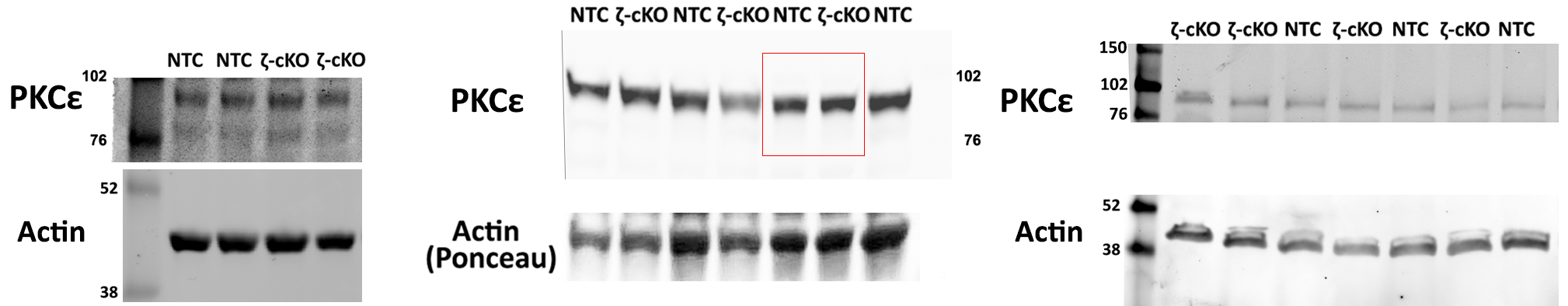

Supplement: Figure 1—source data 2. [file elife-110499-fig1-data2.zip › Figure 1B-Labeled/epsilon/Composite PKCepsilon Actin-Labeled.tif]

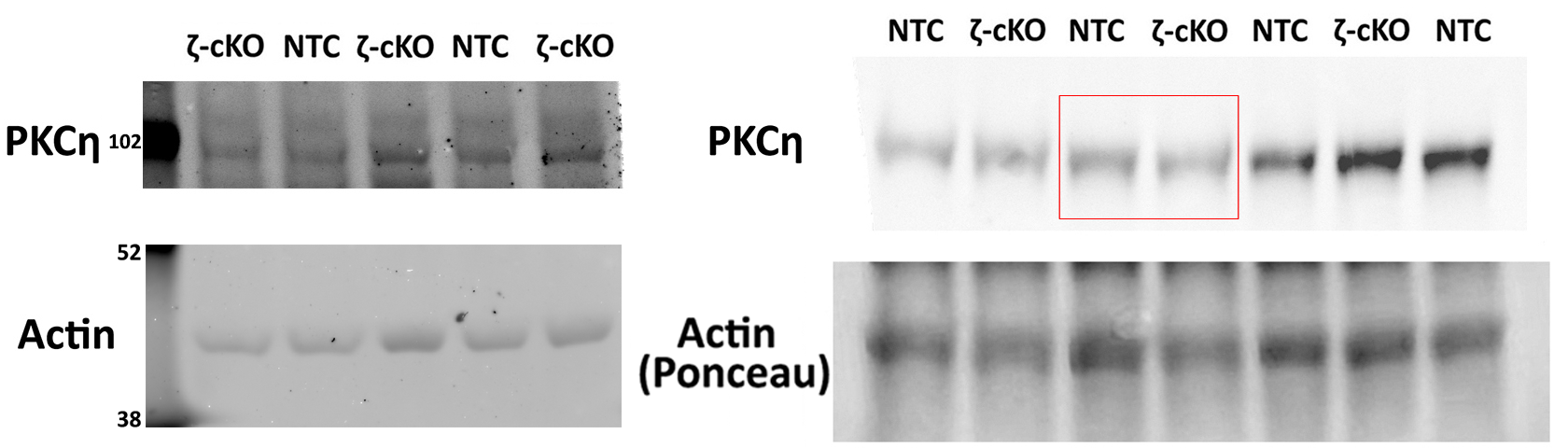

Supplement: Figure 1—source data 2. [file elife-110499-fig1-data2.zip › Figure 1B-Labeled/eta/Composite PKCeta Actin-Labeled.tif]

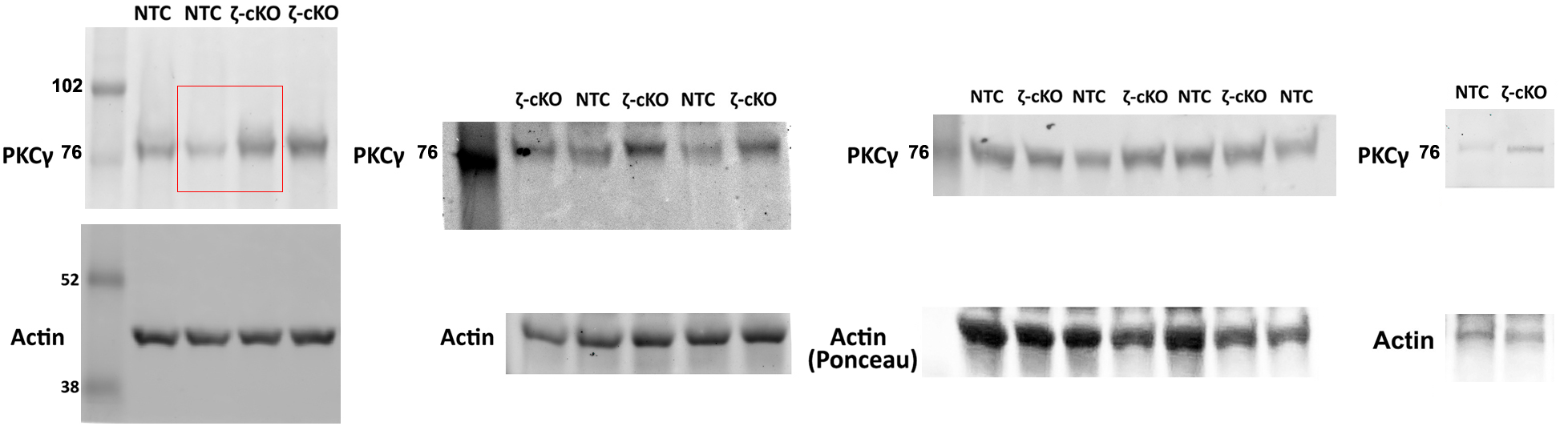

Supplement: Figure 1—source data 2. [file elife-110499-fig1-data2.zip › Figure 1B-Labeled/gamma/Composite PKCgamma Actin-Labeled.tif]

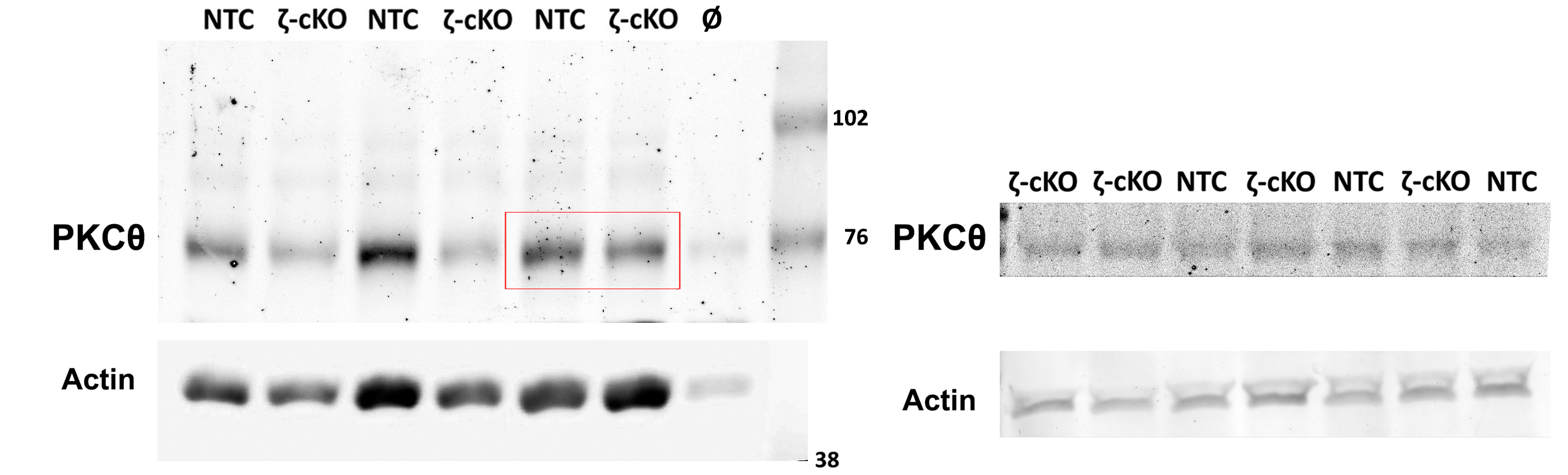

Supplement: Figure 1—source data 2. [file elife-110499-fig1-data2.zip › Figure 1B-Labeled/theta/Composite PKCtheta Actin-Labeled.tif]

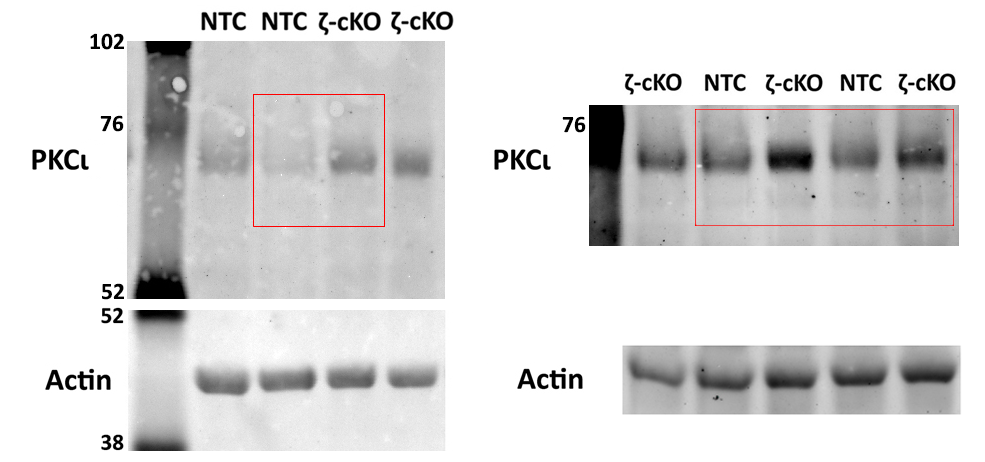

Supplement: Figure 1—source data 2. [file elife-110499-fig1-data2.zip › Figure 1A-Labeled/iota-lambda/Composite PKCiota Actin-Labeled.tif]

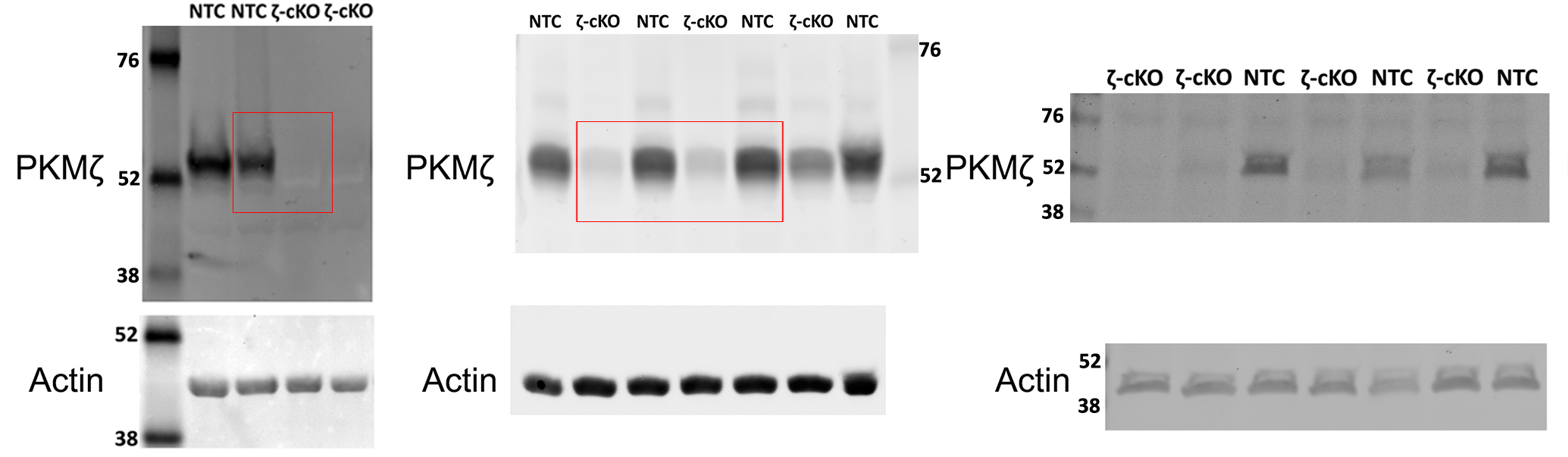

Supplement: Figure 1—source data 2. [file elife-110499-fig1-data2.zip › Figure 1A-Labeled/zeta/Composite PKMzeta Actin-Labeled.tif]

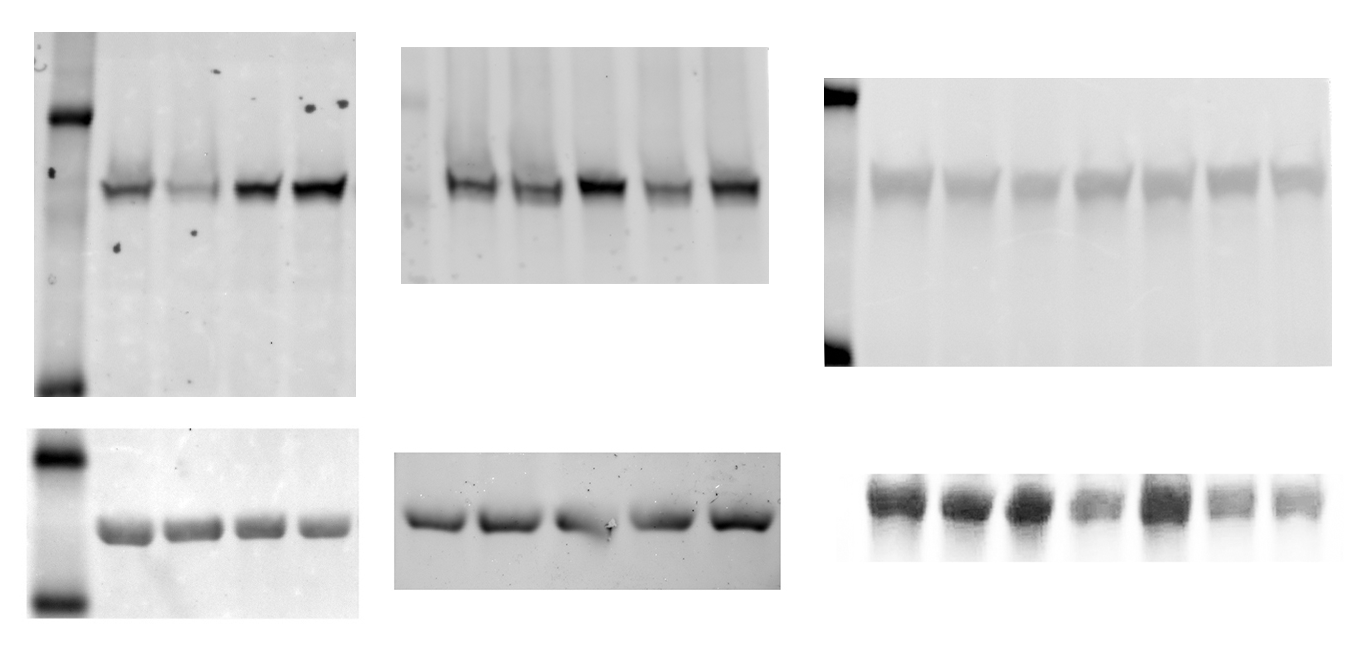

Supplement: Figure 1—source data 3. [file elife-110499-fig1-data3.zip › Figure 1B-Raw/alpha/Raw Data PKCalpha Actin.tif]

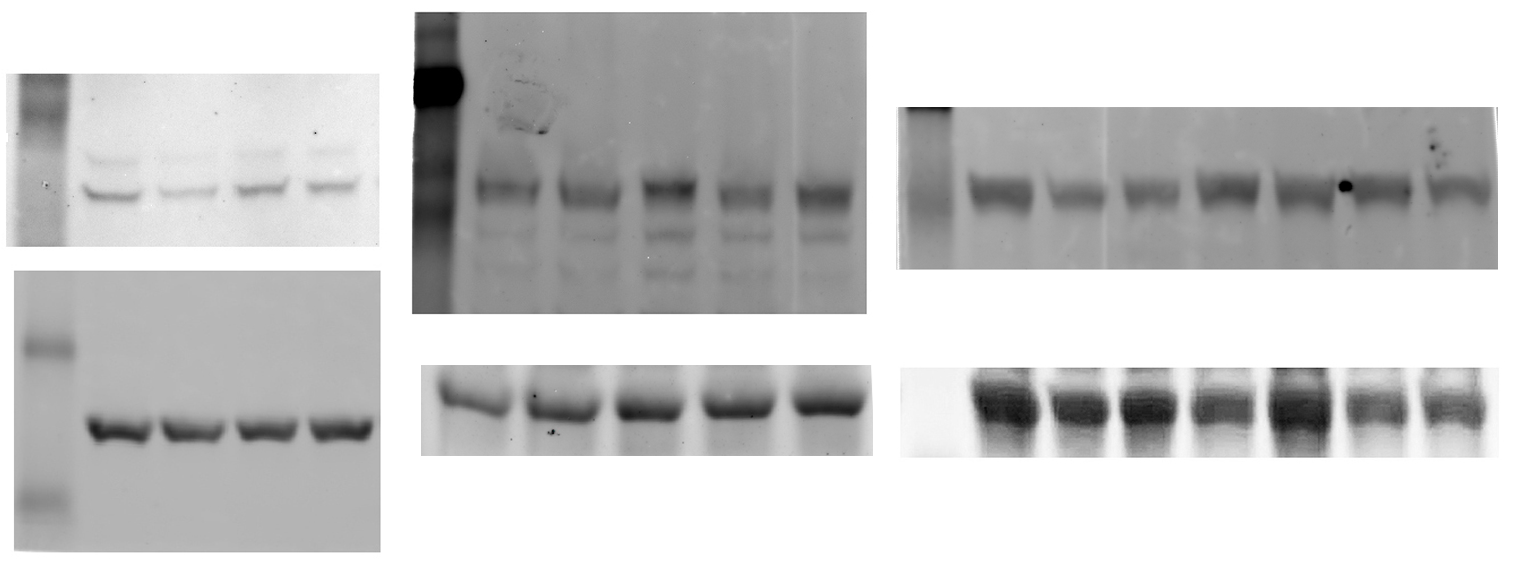

Supplement: Figure 1—source data 3. [file elife-110499-fig1-data3.zip › Figure 1B-Raw/beta I/Raw Data PKCbetaI.tif]

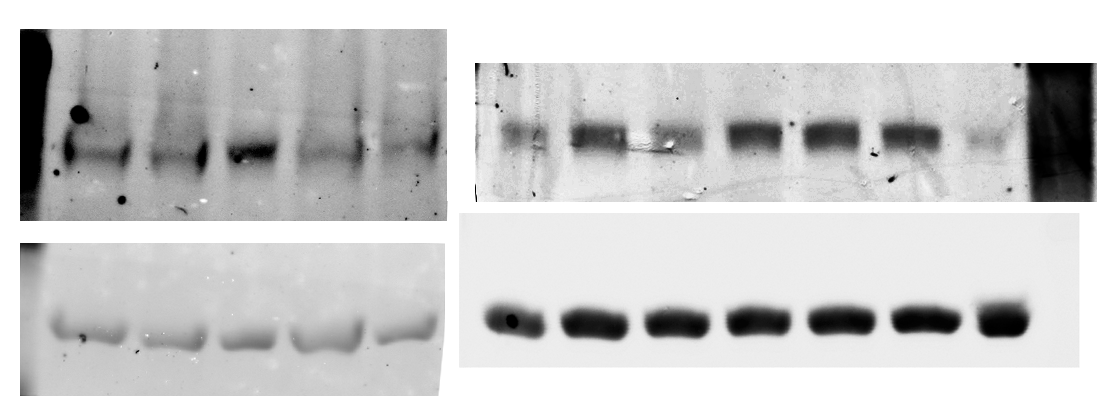

Supplement: Figure 1—source data 3. [file elife-110499-fig1-data3.zip › Figure 1B-Raw/beta II/Raw Data PKCbetaII.tif]

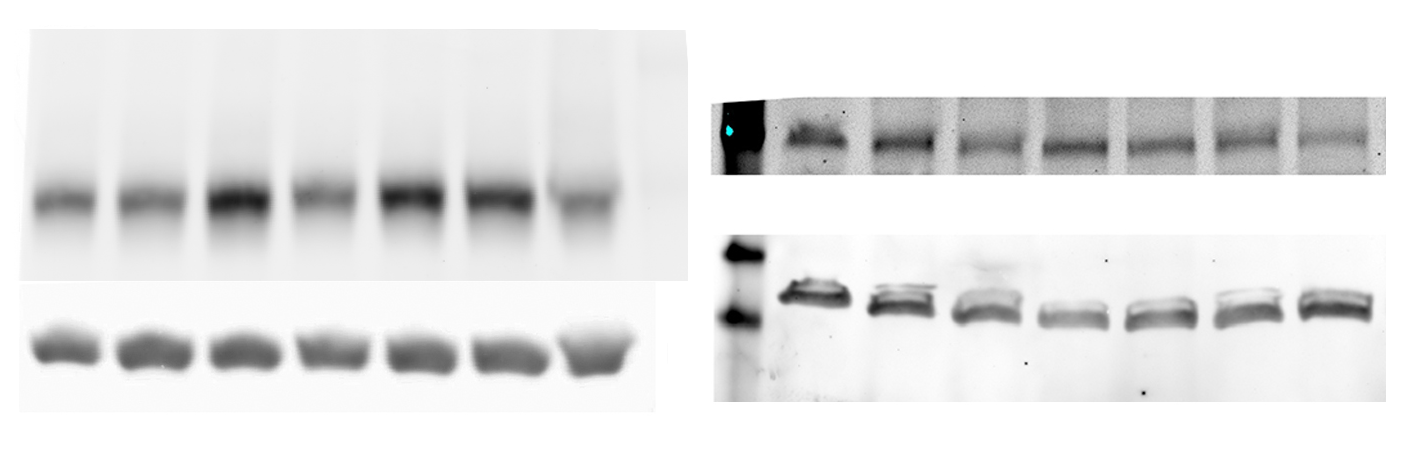

Supplement: Figure 1—source data 3. [file elife-110499-fig1-data3.zip › Figure 1B-Raw/delta/Raw Data PKCdelta.tif]

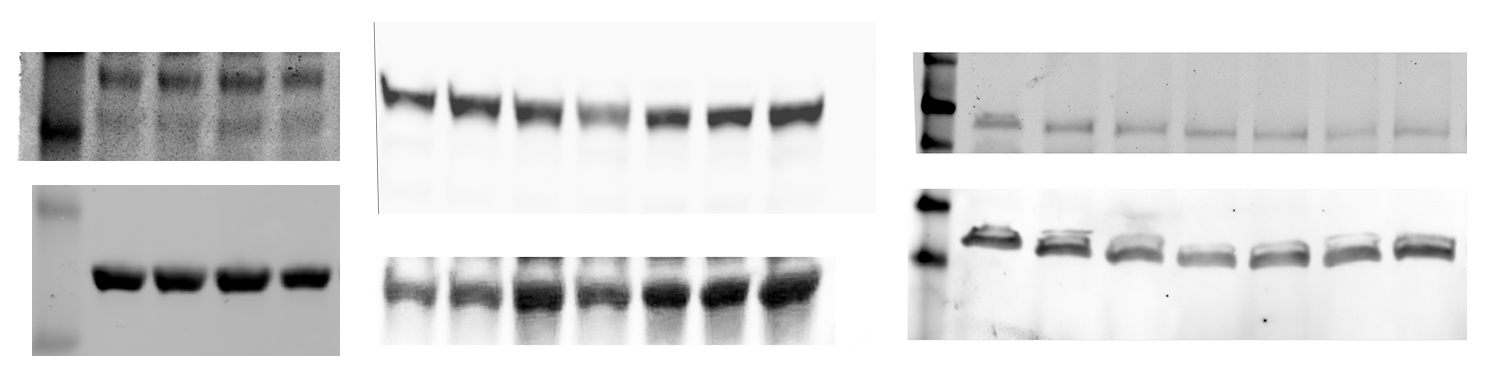

Supplement: Figure 1—source data 3. [file elife-110499-fig1-data3.zip › Figure 1B-Raw/epsilon/Raw Data PKCepsilon.tif]

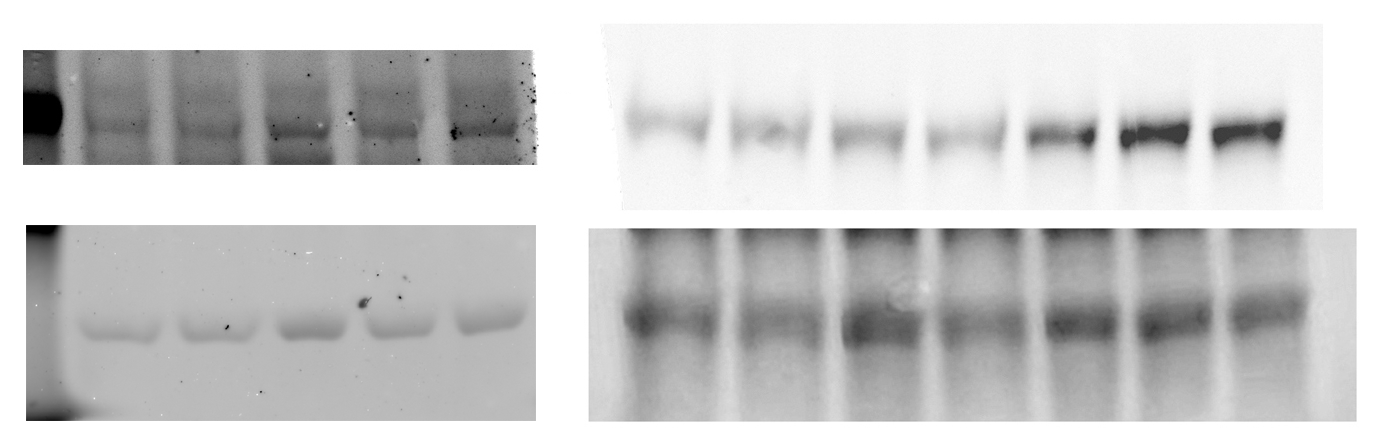

Supplement: Figure 1—source data 3. [file elife-110499-fig1-data3.zip › Figure 1B-Raw/eta/Raw Data PKCeta.tif]

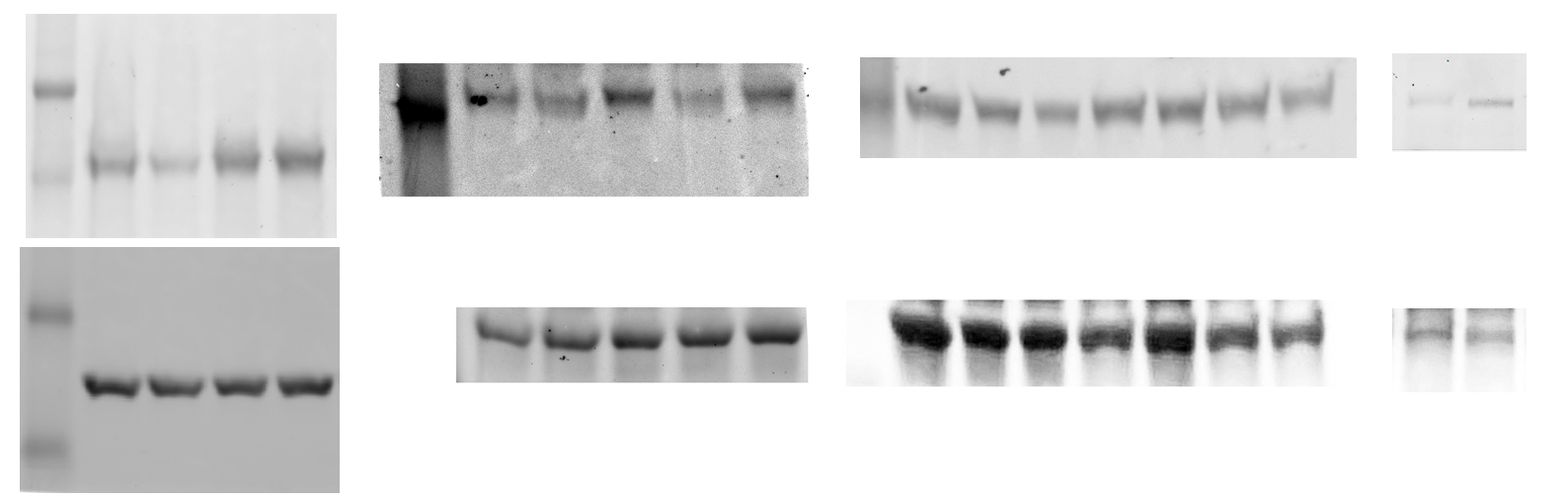

Supplement: Figure 1—source data 3. [file elife-110499-fig1-data3.zip › Figure 1B-Raw/gamma/Raw Data PKCgamma.tif]

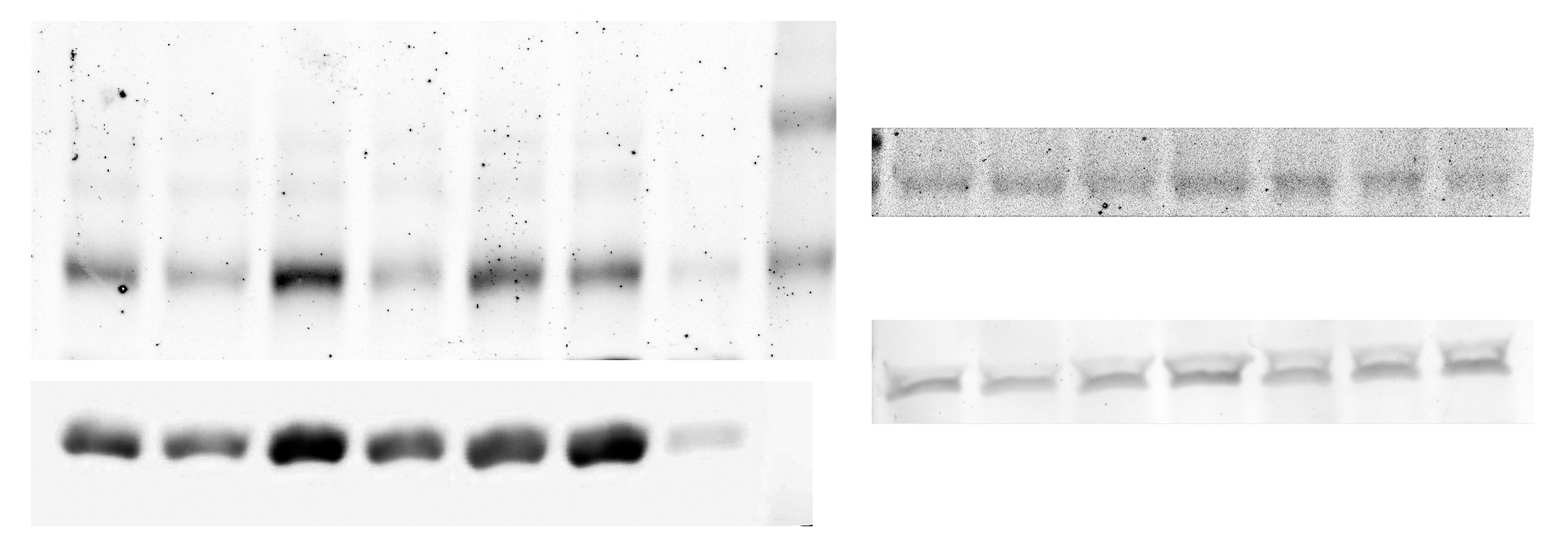

Supplement: Figure 1—source data 3. [file elife-110499-fig1-data3.zip › Figure 1B-Raw/theta/Raw Data PKCtheta.tif]

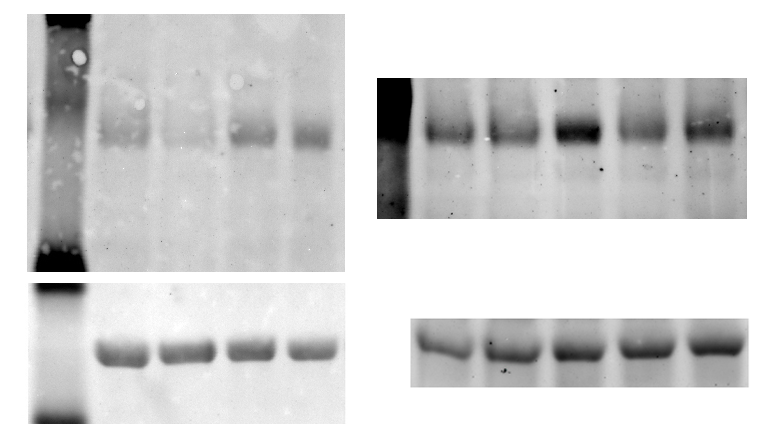

Supplement: Figure 1—source data 3. [file elife-110499-fig1-data3.zip › Figure 1A-Raw/iota-lambda/Raw Data PKCiota.tif]

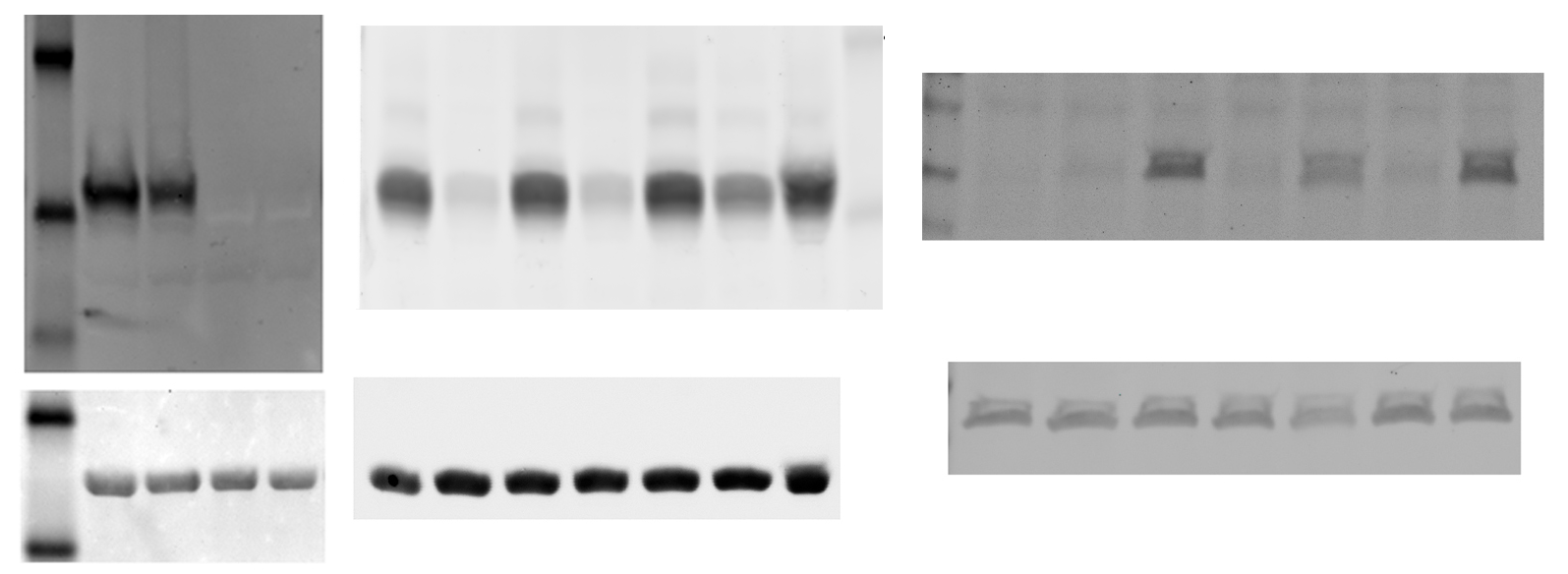

Supplement: Figure 1—source data 3. [file elife-110499-fig1-data3.zip › Figure 1A-Raw/zeta/Raw Data PKMzeta.tif]

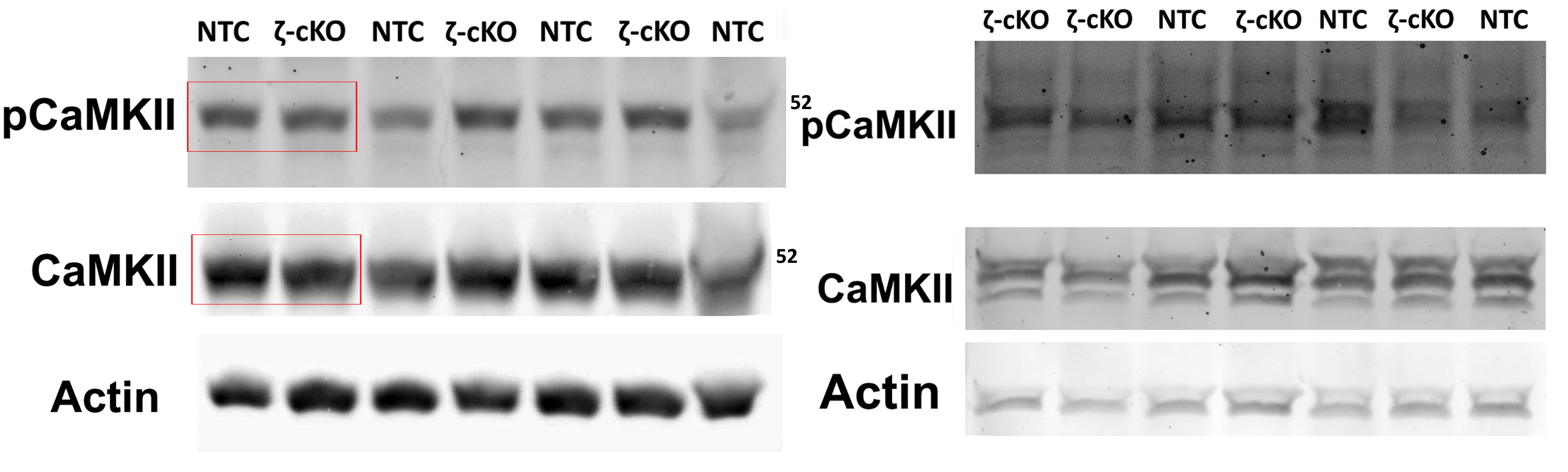

Supplement: Figure 1—figure supplement 1—source data 2. [file elife-110499-fig1-figsupp1-data2.zip › Figure 1-figure supplement 1B-Labeled/pCaMKII CaMKII/Composite pCaMKII_CaMKII_Actin-Labeled.tif]

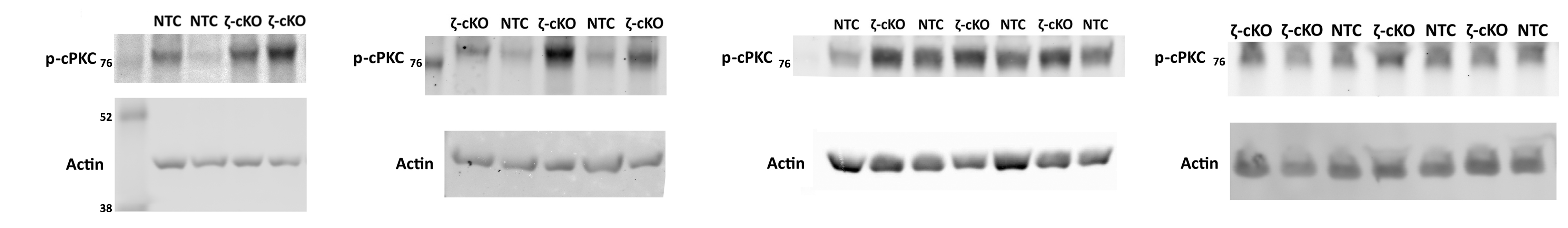

Supplement: Figure 1—figure supplement 1—source data 2. [file elife-110499-fig1-figsupp1-data2.zip › Figure 1-figure supplement 1A-Labeled/pT410 conventional PKC/Composite pT410conventionalPKC Actin-Labeled.tif]

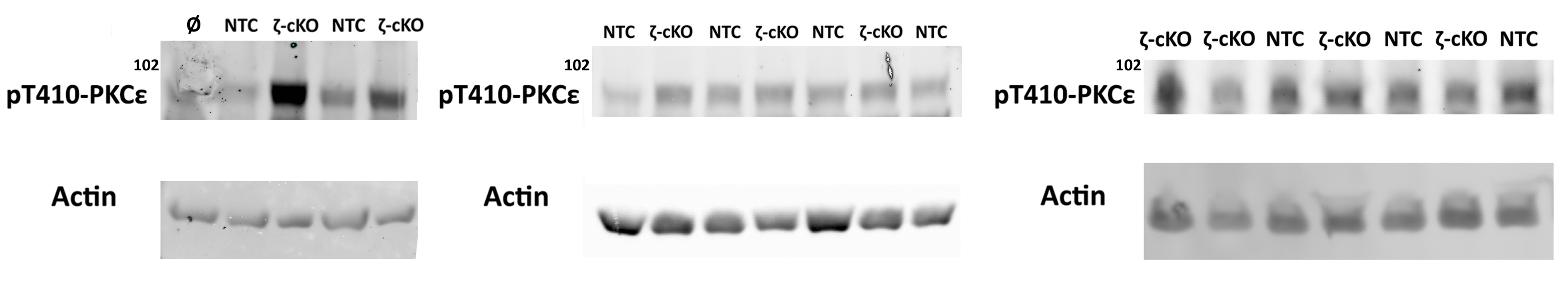

Supplement: Figure 1—figure supplement 1—source data 2. [file elife-110499-fig1-figsupp1-data2.zip › Figure 1-figure supplement 1A-Labeled/pT410 PKCepsilon/Composite pT410PKCepsilon Actin-Labeled.tif]

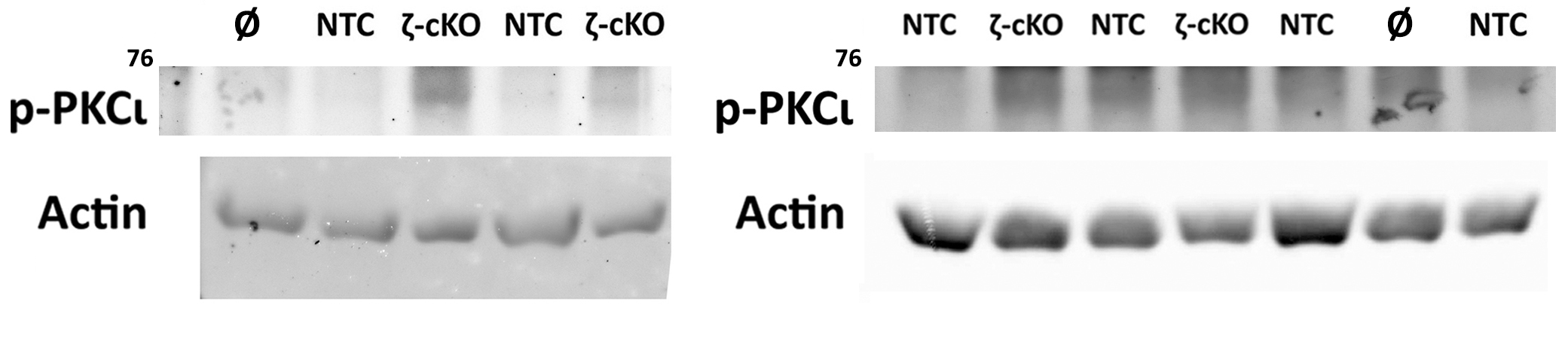

Supplement: Figure 1—figure supplement 1—source data 2. [file elife-110499-fig1-figsupp1-data2.zip › Figure 1-figure supplement 1A-Labeled/pT410 PKCiota-lambda/Composite pT410-PKCiota-lambda Actin-Labeled.tif]

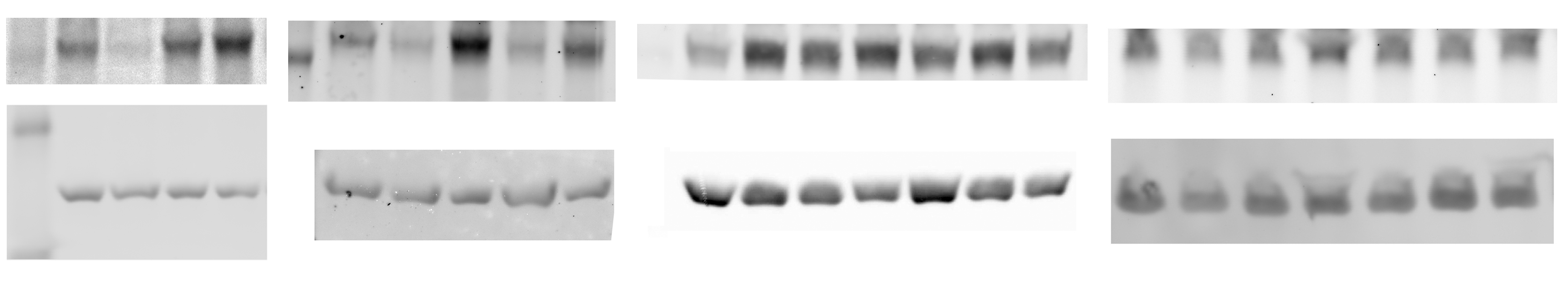

Supplement: Figure 1—figure supplement 1—source data 3. [file elife-110499-fig1-figsupp1-data3.zip › Figure 1-figure supplement 1A-Raw/pT410 conventional PKC/Raw Data pT410conventionalPKC.tif]

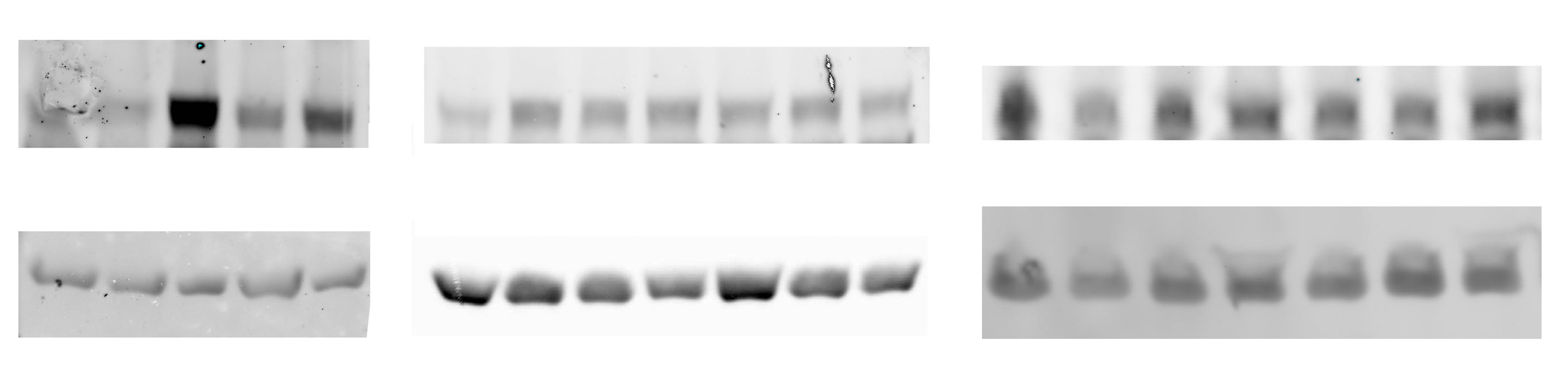

Supplement: Figure 1—figure supplement 1—source data 3. [file elife-110499-fig1-figsupp1-data3.zip › Figure 1-figure supplement 1A-Raw/pT410 PKCepsilon/Raw Data pT410PKCepsilon.tif]

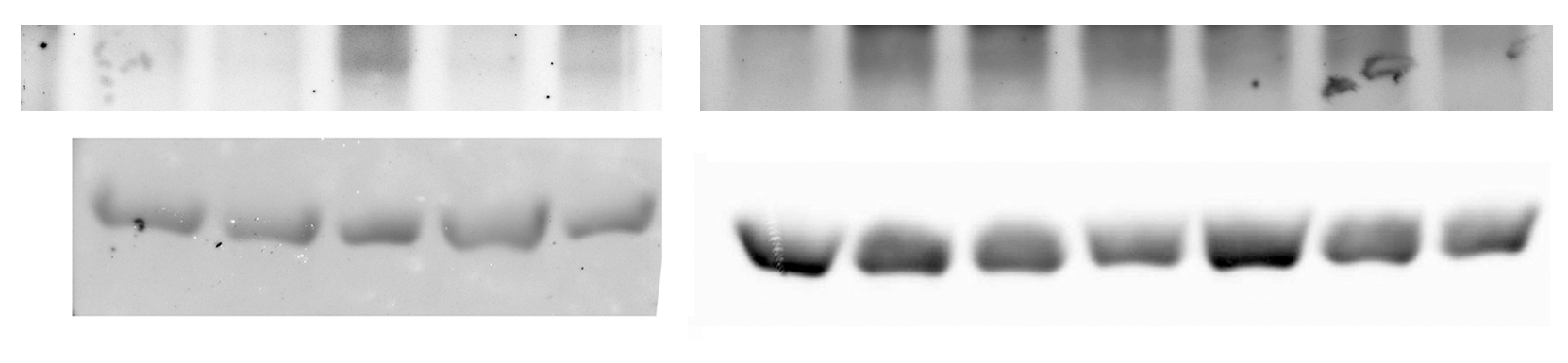

Supplement: Figure 1—figure supplement 1—source data 3. [file elife-110499-fig1-figsupp1-data3.zip › Figure 1-figure supplement 1A-Raw/pT410 PKCiota-lambda/Raw Data pT410-PKCiota-lambda.tif]

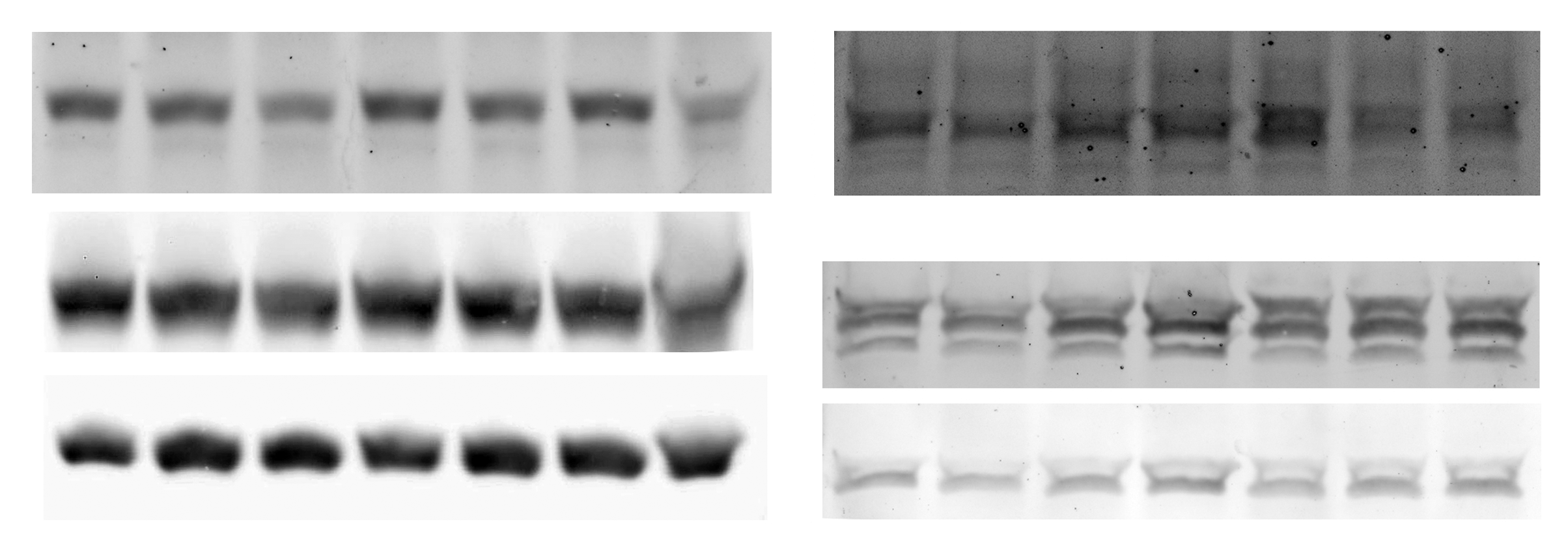

Supplement: Figure 1—figure supplement 1—source data 3. [file elife-110499-fig1-figsupp1-data3.zip › Figure 1-figure supplement 1B-Raw/pCaMKII CaMKII/Raw Data pCaMKII_CaMKII.tif]
