# Supplementary material for: PKMζ-PKCι/λ double-knockout demonstrates atypical PKC is crucial for the persistence of hippocampal LTP and spatial memory
Source: eLife. 2026 Jul 22;15:RP110499. doi: 10.7554/eLife.110499 (PMC13391083; doi:10.7554/eLife.110499)
Supplement: Figure 1—figure supplement 1—source data 1. [file elife-110499-fig1-figsupp1-data1.docx]

|  | Non-transgenic control (NTC) | |  | ζ-cKO | |  |  |  |  |
| --- | --- | --- | --- | --- | --- | --- | --- | --- | --- |
| Kinase | Mean ± S.E.M. | n |  | Mean ± S.E.M. | n | Degree of freedom | *t* | *P* | Cohen’s *d* |
| (**A**) |  |  |  |  |  |  |  |  |  |
| p-PKC |  |  |  |  |  |  |  |  |  |
| **p-PKCι** | **100 ± 11.5** | **6** |  | **189.9 ± 26.6** | **4** | **8** | **3.5** | **0.008** | **2.3** |
| **p-cPKC** | **100 ± 7.8** | **11** |  | **172.9 ± 21.5** | **11** | **20** | **3.2** | **0.005** | **1.4** |
| p-PKCε | 100 ± 8.0 | 9 |  | 127.5 ± 18.0 | 8 | 15 | 1.5 | 0.2 | 0.7 |
| (**B**) |  |  |  |  |  |  |  |  |  |
| CaMKIIα | 100 ± 2.7 | 7 |  | 96.8 ± 4.7 | 7 | 12 | 0.6 | 0.6 | 0.3 |
| p-CaMKIIα | 100 ± 3.4 | 7 |  | 102.6 ± 8.3 | 7 | 12 | 0.3 | 0.8 | 0.2 |

**Figure 1 — figure supplement 1 — source data 1. Statistics for data presented in (A) Figure 1 — figure supplement 1A and (B) Figure 1 — figure supplement 1B.** Significant differences with Bonferroni correction are in bold.
