# Supplementary material for: PKMζ-PKCι/λ double-knockout demonstrates atypical PKC is crucial for the persistence of hippocampal LTP and spatial memory
Source: eLife. 2026 Jul 22;15:RP110499. doi: 10.7554/eLife.110499 (PMC13391083; doi:10.7554/eLife.110499)
Supplement: Figure 2—source data 1. [file elife-110499-fig2-data1.docx]

|  | Vehicle | |  | OH-TAM | |  |  |  |  |
| --- | --- | --- | --- | --- | --- | --- | --- | --- | --- |
| Isozyme / region | Mean ± SEM | n |  | Mean ± SEM | n | Degree of freedom | *t* | *P* | Cohen’s *d* |
| PKMζ |  |  |  |  |  |  |  |  |  |
| ***pyramidale*** | **100 ± 13.6** | **5** |  | **14.1 ± 2.1** | **5** | **8** | **6.2** | **0.0003** | **3.9** |
| ***radiatum*** | **100 ± 3.6** | **5** |  | **23.6 ± 3.9** | **5** | **8** | **14.4** | **<0.0001** | **9.1** |
| *lac-mol* | 100 ± 22.0 | 5 |  | 54.2 ± 13.2 | 5 | 8 | 1.8 | 0.1 | 1.1 |
|  |  |  |  |  |  |  |  |  |  |
| PKCι |  |  |  |  |  |  |  |  |  |
| ***pyramidale*** | **100 ± 9.8** | **5** |  | **235.3 ± 26.4** | **5** | **8** | **4.8** | **0.001** | **3.0** |
| ***radiatum*** | **100 ± 36.9** | **5** |  | **411.2 ± 26.2** | **5** | **8** | **6.9** | **0.0001** | **4.3** |
| *lac-mol* | 100 ± 40.1 | 5 |  | 226.7 ± 26.7 | 5 | 8 | 2.6 | 0.03 | 1.7 |

**Figure 2 — source data 1. Statistics for data presented in Figure 2B.** Significant differences with Bonferroni correction are in bold.
