# Supplementary material for: PKMζ-PKCι/λ double-knockout demonstrates atypical PKC is crucial for the persistence of hippocampal LTP and spatial memory
Source: eLife. 2026 Jul 22;15:RP110499. doi: 10.7554/eLife.110499 (PMC13391083; doi:10.7554/eLife.110499)
Supplement: Peer review history

# Editor report: eLife Assessment

**DOI:** 10.7554/eLife.110499.3.sa0

**Authors:** Laura L Colgin

**Affiliations:** University of Texas at Austin United States

**Keywords:** Convincing, Important

This **important** study addresses the unresolved and long-debated question of whether atypical protein kinase C is required for the maintenance of synaptic potentiation and long-term memory. The **convincing** results confirm previous findings that persistent activity of PKMζ is required for lasting potentiation of hippocampal synapses and spatial memory. The study also adds new genetic evidence to support the earlier suggestion that enhanced expression of PKCι/λ compensates for the genetic reduction of PKMζ to support synaptic potentiation and memory.

# Referee report: Reviewer #1 (Public review)

**DOI:** 10.7554/eLife.110499.3.sa1

Summary:

The authors convincingly demonstrate that when PKMzeta is genetically deleted from the hippocampus, the related atypical PKC, PKClambda is upregulated and compensates both neurophysiologically and behaviorally for the missing PKMzeta. Specifically, the upregulatiion of PKClambda supports late-phase hippocampal long-term potentiation (L-LTP) and long-term spatial memory in the PKMzeta knockout mice.

Strengths:

The study uses up-to-date transgenic techniques to alter the expression of the two atypical PKCs. The synaptic and behavioral experiments are well-controlled and appear to have been carefully executed.

Weaknesses:

None

# Referee report: Reviewer #2 (Public review)

**DOI:** 10.7554/eLife.110499.3.sa2

Summary:

The authors significantly advance understanding of the role of unconventional PKC's, PKCM𝛇 and PKC𝜄/𝝀 in maintenance of late-phase LTP. Their results help to clarify the interplay between "structural" and "biochemical/enzymatic" mechanisms of LTP and learning in the hippocampus.

Strengths:

A strength is the use of state-of-the-art conditional knock-outs of PKCM𝛇 and PKC𝜄/𝝀 to confirm that PKC𝜄/𝝀 compensates for KO of PKCM𝛇 in the hippocampus to maintain long-term potentiation even when PKCM𝛇 is conditionally knocked out in the adult. The authors use both electrophysiological and behavioral methods to assess the effects of genetic manipulations on late-phase LTP and long-term memory. The authors present an informative discussion of the possible molecular mechanisms that may enable compensation by PKC𝜄/𝝀 for KO of PKCM𝛇 in the hippocampus. They correctly emphasize that the notions of "structural" and "enzymatic" mechanisms for maintenance of LTP are not mutually exclusive. With this publication, the experimental case for a role of PKCM𝛇 in maintenance of late-phase LTP is now quite strong.

Weaknesses:

There are no significant weaknesses.

# Author comment: Author response

**DOI:** 10.7554/eLife.110499.3.sa3

**Authors:** Panayiotis Tsokas, Changchi Hsieh, Alejandro Grau-Perales, Andrew Tcherepanov, Leo Kwok, Laura Rodriguez-Valencia, David A Cano, Kim Allen, Hannah J Smith, Sabina Kubayeva, Benson J Wei, Samuel Sabzanov, Rafael Flores-Obando, Sourav Ghosh, Peter John Bergold, Jerry Rudy, James Cottrell, Andre Fenton, Todd Charlton Sacktor

**Affiliations:** Department of Physiology and Pharmacology, The Robert F. Furchgott Center for Neural and Behavioral Science, State University of New York Downstate Health Sciences University Brooklyn United States; Department of Anesthesiology, State University of New York Downstate Health Sciences University Brooklyn United States; https://ror.org/0041qmd21 Department of Pathology, State University of New York Downstate Health Sciences University Brooklyn United States; Department of Physiology and Pharmacology, The Robert F. Furchgott Center for Neural and Behavioral Science, State University of New York Downstate Health Sciences University Brooklyn United States; Center for Neural Science, New York University New York United States; Department of Physiology and Pharmacology, The Robert F. Furchgott Center for Neural and Behavioral Science, State University of New York Downstate Health Sciences University Brooklyn United States; Center for Neural Science, New York University New York United States; Department of Physiology and Pharmacology, The Robert F. Furchgott Center for Neural and Behavioral Science, State University of New York Downstate Health Sciences University Brooklyn United States; Department of Anesthesiology, State University of New York Downstate Health Sciences University Brooklyn United States; https://ror.org/0041qmd21 College of Medicine, State University of New York Downstate Health Sciences University Brooklyn United States; Department of Physiology and Pharmacology, The Robert F. Furchgott Center for Neural and Behavioral Science, State University of New York Downstate Health Sciences University Brooklyn United States; https://ror.org/0041qmd21 Department of Pathology, State University of New York Downstate Health Sciences University Brooklyn United States; https://ror.org/03we2aj97 Department of Biology, City University of New York-Medgar Evers College Brooklyn United States; Department of Physiology and Pharmacology, The Robert F. Furchgott Center for Neural and Behavioral Science, State University of New York Downstate Health Sciences University Brooklyn United States; https://ror.org/01nzkd566 Currently, Department of Neuroscience and Cell Biology, Rutgers University New Brunswick United States; https://ror.org/0041qmd21 College of Medicine, State University of New York Downstate Health Sciences University Brooklyn United States; Department of Physiology and Pharmacology, The Robert F. Furchgott Center for Neural and Behavioral Science, State University of New York Downstate Health Sciences University Brooklyn United States; https://ror.org/0041qmd21 College of Medicine, State University of New York Downstate Health Sciences University Brooklyn United States; Department of Physiology and Pharmacology, The Robert F. Furchgott Center for Neural and Behavioral Science, State University of New York Downstate Health Sciences University Brooklyn United States; Departments of Neurology and Pharmacology, Yale University New Haven United States; Department of Physiology and Pharmacology, The Robert F. Furchgott Center for Neural and Behavioral Science, State University of New York Downstate Health Sciences University Brooklyn United States; Department of Neurology, State University of New York Downstate Health Sciences University Brooklyn United States; https://ror.org/02ttsq026 Department of Psychology and Neuroscience, University of Colorado at Boulder Boulder United States; Department of Anesthesiology, State University of New York Downstate Health Sciences University Brooklyn United States; Department of Physiology and Pharmacology, The Robert F. Furchgott Center for Neural and Behavioral Science, State University of New York Downstate Health Sciences University Brooklyn United States; Center for Neural Science, New York University New York United States; https://ror.org/005dvqh91 Neuroscience Institute at NYU Langone Medical Center New York United States; Department of Physiology and Pharmacology, The Robert F. Furchgott Center for Neural and Behavioral Science, State University of New York Downstate Health Sciences University Brooklyn United States; Department of Anesthesiology, State University of New York Downstate Health Sciences University Brooklyn United States; Department of Neurology, State University of New York Downstate Health Sciences University Brooklyn United States

The following is the authors’ response to the original reviews.

**Public Reviews:**

**Reviewer #1 (Public review):**

Summary:

An ongoing controversy in the field of learning and memory is the specific neural mechanism that maintains long-term memory (LTM). A prominent hypothesis proposed by Sacktor and Fenton and their colleagues is that LTM is maintained by the ongoing activity of the atypical PKC isoform PKMζ. Early evidence in support of this hypothesis came from experiments showing that an inhibitory peptide, ZIP, whose activity was purported to be specific for PKMζ, blocked late-phase hippocampal LTP (L-LTP) and LTM. However, in 2013, two articles reported that LTM was normal in PKMζ knockout mice and that ZIP erased LTM in the knockout mice, indicating that ZIP lacked specificity for PKMζ. In response, Sacktor and Fenton and colleagues reported in 2016 that in PKMζ null mice, there is an increase in the expression of PKC𝜾/𝝀, a related isoform of atypical PKC, and this increased expression can compensate for PKMζ; their data indicated that the upregulation of PKC𝜾/𝝀 mediates L-LTP and LTM in the PKMζ. In the present article, the authors provide additional support for this idea. They replicate the finding of an upregulation of PKC𝜾/𝝀 expression in the hippocampus of PKMζ knockout mice; in addition, they show that the expression of several other PKC isoforms is upregulated in the knockouts. They find that down-regulation of PKC𝜾/𝝀 expression in the hippocampus using the Cre-LoxP technology, the 2016 paper merely used an inhibitor to block the activity of PKC𝜾/𝝀-blocks L-LTP. Finally, the authors demonstrate that, although LTM is preserved in the single PKMζ knockout mouse, it is eliminated in the PKMζ/PKC𝜾/𝝀 double knockout mouse.

Strengths:

The experiments appear to have been carefully executed, the results reliable, and the paper well-written. Overall, the article provides significant additional support for the idea that the activity of PKMζ is critical for the maintenance of hippocampal L-LTP and LTM. The article uses genetic methods, rather than simply pharmacological ones, to demonstrate that when PKMζ is genetically deleted, PKC𝜾/𝝀, compensates for the missing PKCζ.

Weaknesses:

The paper sets up what I believe is probably a false dichotomy between a structural explanation - a change in the number of synaptic connections among neurons - and the persistent kinase activity explanation for memory maintenance. Why are these two explanations necessarily antithetical? It is possible that an increase in synaptic connections and the ongoing activity of PKMζ both contribute substantially to memory maintenance. The authors certainly don't provide any evidence that the number of synapses in the hippocampus remains unchanged after the induction of L-LTP or LTM. Indeed, I see no reason why persistent PKMζ activity could not be a mechanism for the maintenance of an enhanced number of synaptic connections following the induction of LTP/LTM. To the best of my knowledge, this possibility has not yet been explored. Consequently, I don't see why the present results would lead one to favor a biochemical explanation over a structural one for memory maintenance. Given the significant experimental evidence that LTM involves persistent structural changes in neurons, both explanations are equally plausible at present.

As requested, we eliminated the discussion of a dichotomy between structural and biochemical mechanisms of long-term memory in the Abstract and Introduction. We now briefly address the relationship between the two hypotheses, which are not mutually exclusive, in the Discussion.

**Reviewer #2 (Public review):**

Summary:

The authors are attempting to advance understanding of the role of unconventional PKCs, PKCM𝛇, and PKC𝜄/𝝀 in maintenance of late-phase LTP. Their results help to clarify the interplay between "structural" and "biochemical/enzymatic" mechanisms of LTP and learning in the hippocampus.

Strengths:

A strength is the use of conditional knock-outs of PKCM𝛇 and PKC𝜄/𝝀 to assess the role of these two enzymes in maintaining long-term potentiation and in compensating for each other when one of them is conditionally knocked out in the adult.

Weaknesses:

The paper is extremely difficult to read because the abstract does not clearly state the advances made over earlier studies by the use of conditional KO mutation. For example, in line nine of the abstract, the authors state, "Here, we found PKC𝜄/𝝀 persists in LTP and long-term memory when PKM𝛇 is genetically deleted." This is confusing because it sounds as though the experiments have repeated earlier published experiments in which the gene encoding PKM𝛇 is deleted in the embryo. The authors are not clear throughout the manuscript that they are using conditional KO of the two enzymes in the adult animal, rather than deletion of the gene. The term "genetically deleted" does not mean "conditionally deleted in the adult." The final sentences of the abstract are: "Whereas deleting PKM𝛇 and PKC𝜄/𝝀 individually induces compensation, deleting both aPKCs abolishes hippocampal late-LTP. Hippocampal 𝜄/𝝀-𝛇 -double-knockout eliminates spatial long-term memory but not short-term memory. Thus, in the absence of PKM𝛇 , a second persistent biochemical process compensates to maintain late-LTP and long-term memory." These sentences do not convey a clear logical conclusion. The Discussion does a better job of stating the importance of the experiments.

We have clarified the genotypes of the mice in the abstract and throughout the text.

**Reviewer #3 (Public review):**

Summary:

The manuscript addresses an important, yet unresolved and long-debated, question: whether atypical protein kinase C is required for the maintenance of late-long-term synaptic potentiation (L-LTP) and long-term memory (LTM). The authors confirm previous findings that persistent activity of PKMζ is required for hippocampal L-LTP and spatial memory. They demonstrate that genetically deleting PKCι/λ and PKMζ individually induces compensatory upregulation, whereas deleting both atypical PKCs abolishes hippocampal L-LTP spatial long-term memory. The study uses an elegant combination of immunoblots, electrophysiology, and behavioral assays. The use of Cre-recombinase to target specific hippocampal regions and neurons adds to the rigor of the findings.

Strengths:

The manuscript addresses an important, yet unresolved and long-debated, question; whether PKMζ is required for the maintenance of L-LTP and LTM. The study demonstrates that PKCι/λ, which was previously shown to be critical for the initial generation of the early phase of LTP and short-term memory, becomes persistently active in L-LTP and LTM in a PKMζ knock-out model, compensating for the loss of PKMζ. Furthermore, when the compensation mechanisms are eliminated by simultaneous deletion of both PKMζ and PKCι/λ, maintenance of LTP and long-term spatial memory, but not of short-term memory, is diminished. The strength of this study is that the authors used a double-knockout strategy to directly address the controversy concerning the roles of PKMζ in memory formation. By showing that PKCι/λ compensates when PKMζ is deleted, the authors provided a compelling explanation for previous contradictory findings.

Weaknesses:

(1) The authors should provide the numerical values for all data.

(2) It appears that blind procedures were only used for the behavioral experiments. Some explanation is warranted.

(3) The description of the immunoblotting procedures lacks sufficient detail. The authors state that immunoblots were stained with multiple antisera to visualize multiple PKCs on the same immunoblot. To conserve antisera, the immunoblots were cut to isolate the relevant proteins based on molecular weight. Isoforms with similar molecular weights were either stained with antisera of different species or on separate blots. Despite this explanation, it is unclear how immunoblotting was performed in practice. For example, in Figure 1B, the authors compared the changes of four conventional PKC isoforms. Because all four antibodies are mouse monoclonal antibodies recognizing proteins of similar molecular weights, each probing should presumably have its own actin loading controls. However, these controls are missing from the figure. Some clarification is warranted.

(4) The statement in the legend to Figure 4B, that the increases of maximum avoidance time from pretraining to trial 1 are not different, indicates both groups of mice successfully established short-term memory, which is not correct. The analysis only reveals that there is no difference between the two groups. No differences could be due to both groups learning the same, as the authors suggest, or alternatively to no learning in either group.

(5) The labeling on some of the illustrations (e.g., Figure 2B) is unreadable.

(6) In Figure 4B, only the single statistical comparison between "pretaining" and "1 trial" is shown. The other comparisons described in the legend should also be illustrated.

(7) There is no documentation to support the statement that "The prevailing textbook mechanism for how memory is retained asserts that stable structural changes at synapses, the result of initial protein synthesis and growth, sustain memory without the need for ongoing biochemical activity dedicated to storing information" or for the statement in the Discussion that the structural model of memory storage is the standard account.

(1) Numerical data used in statistical analyses are now provided for LTP and all other experiments in the Numerical data file.

(2) Blind procedures were performed for all experiments except for LTP experiments that involved the transfection of eGFP as control, as the eGFP could be detected visually in the hippocampal slice by the experimenter. This is now clarified in the Statistics section of the Methods.

(3) The description of immunoblotting was clarified in the Methods, and actin loading controls presented for all immunoblots in Figure 1 and Figure 1—figure supplements 1 and 2, as well as the source data files for these figures showing the original immunoblots.

(4) Short-term memory (Figure 5B) is now determined by 2 methods. First, we show that for both groups the times to enter the shock zone increase in the first training trial, as compared to the pretraining session with the shock off. The increases are not different between the groups. Second, we show increases of the maximal avoidance time from pretraining to trial 1 for both groups are significant, and that the increases are not different. These data show that short-term memory was present in both groups and not measurably different between the groups.

(5) The fonts of the figure labels were enlarged.

(6) The comparisons between pretraining and training trial 1 and between training trials 1 and 3 for the two groups are now shown in Figure 5B.

(7) We abbreviated our discussion of the structural model, which is now presented at the end of the Discussion (as per Reviewer 1), and removed the comment that it is the prevailing view, stating instead that the hypothesis is “widely held.”

Additional points: As requested, the timing of tamoxifen injections and tissue collection for immunohistochemistry is clarified in the protocol schematic of a new Figure 2A and Figure 2A legend.
